# The future of neuropsychology is digital, theory-driven, and Bayesian: a paradigmatic study of cognitive flexibility

**DOI:** 10.3389/fpsyg.2024.1437192

**Published:** 2024-07-12

**Authors:** Clara Schmerwitz, Bruno Kopp

**Affiliations:** Cognitive Neuropsychology, Department of Neurology, Hannover Medical School, Hannover, Germany

**Keywords:** digital neuropsychology, online assessment, theory-driven research, Bayesian statistics, executive function, cognitive flexibility, Wisconsin card sorting, perseveration errors

## Abstract

**Introduction:**

This study explores the transformative potential of digital, theory-driven, and Bayesian paradigms in neuropsychology by combining digital technologies, a commitment to evaluating theoretical frameworks, and Bayesian statistics. The study also examines theories of executive function and cognitive flexibility in a large sample of neurotypical individuals (*N* = 489).

**Methods:**

We developed an internet-based Wisconsin Card-Sorting Task (iWCST) optimized for online assessment of perseveration errors (PE). Predictions of the percentage of PE, PE (%), in non-repetitive versus repetitive situations were derived from the established supervisory attention system (SAS) theory, non-repetitive PE (%) < repetitive PE (%), and the novel goal-directed instrumental control (GIC) theory, non-repetitive PE (%) > repetitive PE (%).

**Results:**

Bayesian *t*-tests revealed the presence of a robust error suppression effect (ESE) indicating that PE are less likely in repetitive situations than in non-repetitive situations, contradicting SAS theory with posterior model probability *p* < 0.001 and confirming GIC theory with posterior model probability *p* > 0.999. We conclude that repetitive situations support cognitive set switching in the iWCST by facilitating the retrieval of goal-directed, instrumental memory that associates stimulus features, actions, and outcomes, thereby generating the ESE in neurotypical individuals. We also report exploratory data analyses, including a Bayesian network analysis of relationships between iWCST measures.

**Discussion:**

Overall, this study serves as a paradigmatic model for combining digital technologies, theory-driven research, and Bayesian statistics in neuropsychology. It also provides insight into how this integrative, innovative approach can advance the understanding of executive function and cognitive flexibility and inform future research and clinical applications.

## 1 Introduction

Neuropsychology needs innovation (Casaletto and Heaton, 2017; Loring et al., 2018; Parsons et al., 2018; Bilder and Reise, 2019; Kessels, 2019). In this article, we propose three main pillars for the necessary transformation: digitization, theory-driven research, and Bayesian statistics. The empirical study presented here illustrates how these three innovative trends can be combined to advance neuropsychology. It provides an example of how the incorporation of digital tools; the explicit formulation of hypotheses based on existing theories and the use of these theories to guide assessment design, data collection, and analysis; and the application of Bayesian methods to the statistical analysis of neuropsychological data provide opportunities for the future development of the discipline.

**Figure 1:**
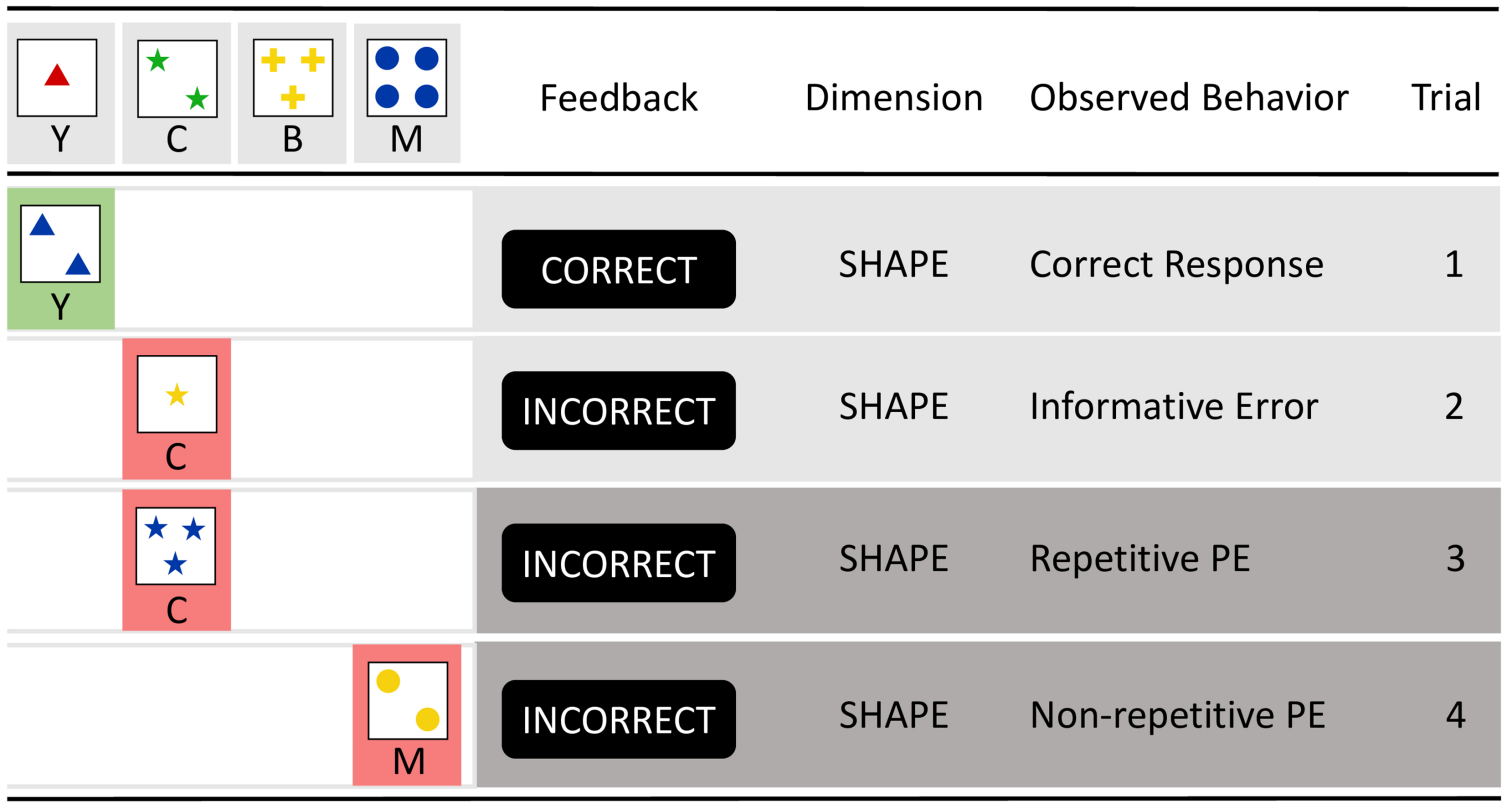
The figure on the left shows the typical layout of computerized Wisconsin card-sorting tasks. Wisconsin stimulus cards can be described in a three-dimensional space (with the dimensions COLOR/SHAPE/NUMBER of the items represented). Each dimension is instantiated by one of four features (with COLOR features: red, green, yellow, blue/SHAPE features: triangle, asterisk, cross, circle/NUMBER features: #1, #2, #3, #4). On each trial, an action is required that matches the current target card to one of four consistently presented keycards (i.e., outer left keycard: ‘1 red triangle’, inner left keycard: ‘2 green asterisks’, inner right keycard: ‘3 yellow crosses’, outer right keycard: ‘4 blue circles’). The task is to select, on each trial, the keycard that shares the feature with the target feature to be prioritized (be it the COLOR, SHAPE, or NUMBER feature). This is illustrated on trial 1 by selecting the left-most keycard ‘1 red triangle’, which shares the SHAPE feature ‘triangle’ with the current target card, by pressing the Y key. The feedback CORRECT indicates that sorting by SHAPE on trial 1 was correct. Trial 2 shows the selection of the inner left keycard ‘2 green asterisks’, which shares the SHAPE feature ‘asterisk’ with the target card, by pressing the C key. However, the feedback INCORRECT indicates that sorting by SHAPE was no longer correct on trial 2; this feedback is therefore an informative error signal. Trials 3 and 4 illustrate perseveration errors (PE) in repetitive vs. non-repetitive situations. Trial 3 shows a repetitive PE in which, despite the informative error signal obtained on trial 2, sorting by SHAPE is applied, and the action-relevant SHAPE feature (asterisks on trials 2 and 3) is repeated on the two successive trials (hence the label *repetitive* PE or rPE). Trial 4 shows a non-repetitive PE, where sorting by SHAPE is applied despite the informative error signal obtained on trial 3, but the action-relevant SHAPE feature (asterisk on trial 3, circle on trial 4) changes between the two successive trials (hence the label *non-repetitive* PE or nPE).

Digital approaches to neuropsychology, including computerized cognitive assessments, especially unsupervised online assessments, are a growing trend in neuropsychological research (Feenstra et al., 2017; Germine et al., 2019; Parsons and Duffield, 2020; Libon et al., 2022). These tools have the potential to provide measures of cognitive function in a variety of settings, and demonstrating their psychometric quality in terms of validity, reliability, and diagnostic utility is an important step in the digital transformation that also offers new opportunities for standardized neuropsychological testing (Brooks et al., 2009; Kiselica et al., 2023). In the present study, we present an internet-based version of the well-known Wisconsin Card Sorting Test (WCST; originally developed by Berg, 1948, and Grant and Berg, 1948), which we refer to as the internet-based Wisconsin Card-Sorting Task (iWCST), and which is described in detail in the Materials and Methods section below (see also Figure 1). Standardized (Heaton, 1981; Heaton et al., 1993; Kongs et al., 2000) and computerized (e.g., Barceló, 2003; Heaton and PAR Staff, 2003a,b; Kopp and Lange, 2013) versions of the WCST, as well as a variety of additional WCST versions (Modified Card Sorting Test, MCST, Nelson, 1976; Modified Wisconsin Card Sorting Test, M-WCST, Schretlen, 2010), are widely used in clinical and research settings. Please note that the computerized versions of the WCST (Heaton and PAR Staff, 2003a,b) are also available as a commercial product for online administration on PARiConnect.^1^

All of these different WCST versions are commonly used to assess executive function and cognitive flexibility in individuals with neurological diseases and psychological disorders. Cognitive flexibility, the ability to switch between different cognitive sets, is a topic of central interest in neuropsychology (see Kopp, in press, for a review). Milner (1963) pioneered this extensive line of WCST-based neuropsychological research on cognitive flexibility, which has been the subject of several reviews and meta-analyses (e.g., Demakis, 2003; Nyhus and Barceló, 2009; Lange et al., 2017, 2018; Miles et al., 2021).

Theory-driven approaches remain critical in science because they provide a framework to guide hypothesis formulation and study design, to better understand data, and to ensure that analyses are theoretically relevant. Theory falsification remains important to scientific inquiry, underscoring its central role in advancing scientific understanding (Popper, 1934/35). However, theory-driven investigations in neuropsychology can be difficult when theoretical frameworks lack precise quantitative predictions that can be rigorously tested. The present study illustrates a method for subjecting neuropsychological theories to falsifiability tests, even in the absence of quantitative predictions, with the goal of providing a way to ultimately distill the most valid theoretical framework.

The Supervisory Attentional System (SAS) theory explains how executive control operates in goal-directed tasks (Norman and Shallice, 1981; Shallice, 1982; Shallice and Burgess, 1991, 1996; Shallice and Cooper, 2011). The SAS plays a critical role in managing and coordinating lower-level cognitive processes, called schemas, to achieve higher-order goals. The SAS is responsible for supervisory control, intervening when the schemas appear inadequate to achieve a goal, for example, after errors and in non-routine situations. Impairments in the SAS can manifest as cognitive inflexibility, which affects an individual’s ability to adapt to changing environmental demands, presumably due to a difficulty in shifting cognitive sets.

As shown in Figure 1, the WCST asks individuals to sort cards according to changing dimensions, such as color, shape, or number. Perseveration errors (PE) occur when individuals continue to apply a dimension that was previously correct but has since become incorrect. PE are thus a behavioral expression of a difficulty in adapting to new environmental demands, presumably indicating an inability to shift cognitive sets (Kopp, in press). From the perspective of SAS theory, the SAS, as an integral part of executive control, is critically involved in monitoring and adjusting cognitive sets. Impairments in the SAS may therefore increase PE percentages in the WCST, highlighting associations between SAS impairments and deficits in cognitive flexibility.

In recent years, we have identified two subtypes of PE that differ in their prevalence, a phenomenon referred to as the error suppression effect (ESE; Kopp et al., 2019, 2023; Steinke et al., 2020a). Within this framework, two distinct types of PE have been recognized: those that occur in non-repetitive situations (referred to as non-repetitive PE or nPE throughout this article) and those that occur in repetitive situations (referred to as repetitive PE or rPE throughout this article), as shown in Figure 1. As can be seen, non-repetitive PE (nPE) occur when the same incorrect response strategy is used despite the provision of informative feedback indicating the need for change, but the relevant feature changes between successive trials. Repetitive PE (rPE) occur when the same incorrect response strategy is repeated despite the provision of this type of informative feedback, but the relevant feature remains constant across successive trials. Importantly, repetitions can be viewed as retrieval cues, as will be discussed later. Non-repetitive situations, which involve changes in both action-relevant features and actions, are non-routine conditions. In contrast, repetitive situations, which involve repetition in both action-relevant features and actions, are routine conditions. Contrary to the prediction of the SAS theory that non-repetitive PE should be less frequent than repetitive PE due to a stronger involvement of the SAS in non-routine situations, our studies led to the identification of the ESE where repetitive PE are less frequent than non-repetitive PE. Initially discovered in a sample of neurological inpatients (Kopp et al., 2019), subsequent studies confirmed the ESE in neurotypical individuals, with estimated effect sizes (Cohen’s *d*) of 0.53 (Steinke et al., 2020a; *N* = 375) and 0.50 (Kopp et al., 2023, Study 1; *N* = 40).

**Figure 2:**
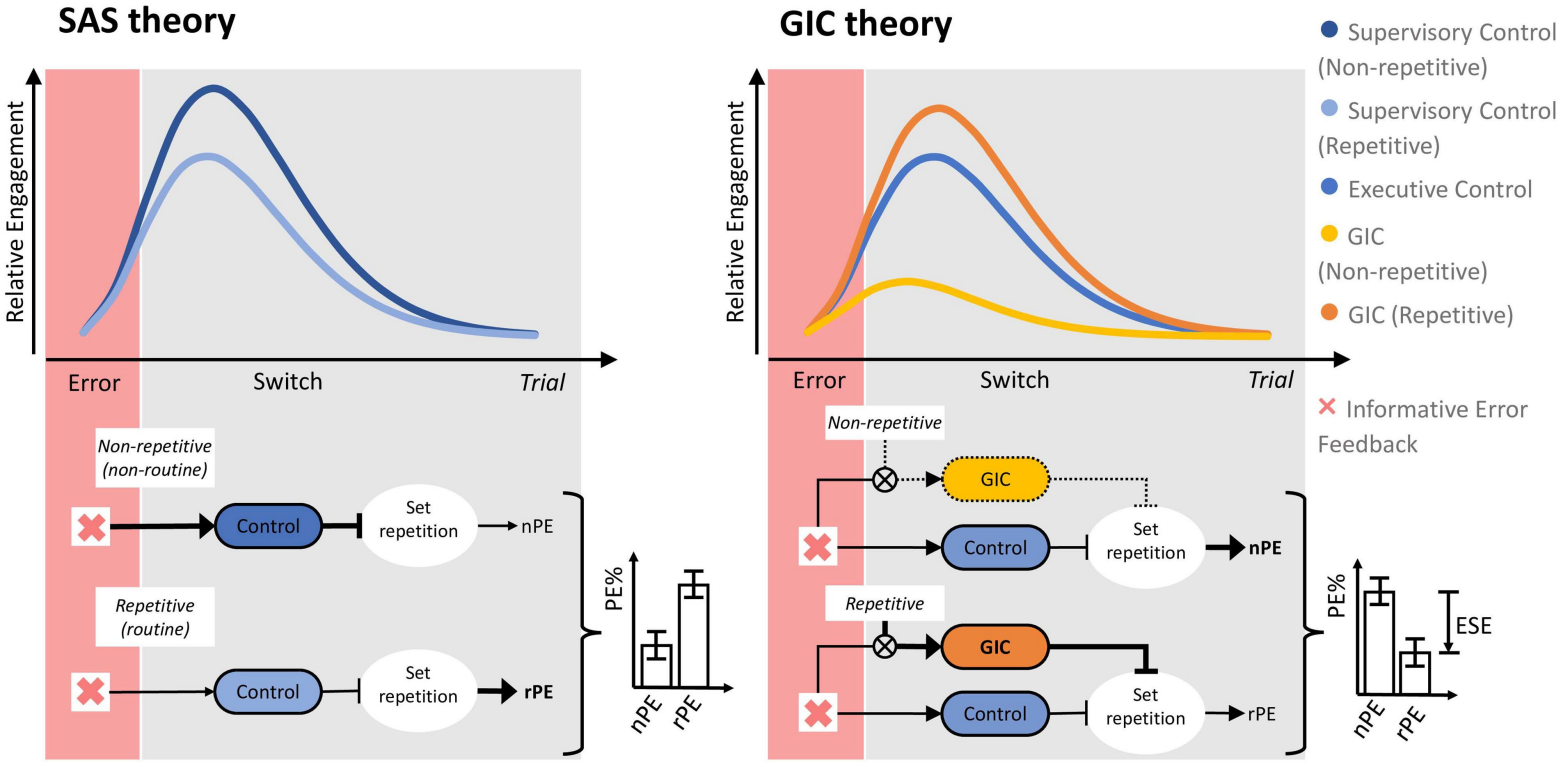
Illustration of the supervisory attentional system (SAS) theory and the goal-directed instrumental control (GIC) theory. The top graphs show the relative engagement (y-axis) of the conceptualized transient control processes as a function of time (x-axis) following an informative error signal. The figure on the left shows that SAS theory suggests that the supervisory control system is more involved in non-repetitive, relatively non-routinized than in repetitive, relatively routinized situations, with the effect that set repetition is more strongly inhibited in non-repetitive than in repetitive situations, leading to the prediction non-repetitive PE (%) < repetitive PE (%). The figure on the right shows that GIC theory proposes dissociable pathways to set inhibition, with feedback-based control activated equally strongly by informative error signals in both non-repetitive and repetitive situations. Retrieval-based control comes into the play primarily on repetitive trials, where feature repetition (see Figure 1) facilitates set switching through retrieving goal-directed, instrumental memory traces that associate action-relevant features, actions, and outcomes. The involvement of this pathway on repetitive trials leads to the prediction of an error suppression effect (ESE), i.e., non-repetitive PE (%) > repetitive PE (%). See Figure 11 for more details on the GIC theory. Arrowheaded lines (↑) indicate facilitative effects, and T-shaped lines (T) indicate inhibitory effects.

In the aforementioned investigation (Kopp et al., 2023), which involved multiple experiments, we found that goal-directed instrumental learning (Dickinson, 1994; De Wit and Dickinson, 2009; Balleine and O’Doherty, 2010) plays a central role in generating the ESE. We developed the theory of goal-directed instrumental control (GIC), according to which repetitive conditions for PE facilitate the retrieval of goal-directed instrumental memory. These memory traces include associative bindings between action-relevant features, actions and their outcomes on recent WCST trials. As a result of retrieving these goal-directed, instrumental memory traces, which on switch trials include bindings between action-relevant features, actions, and negative outcomes (i.e., error feedback), repetitive PE should be less frequent than non-repetitive PE.^2^ Taken together, the manifestation of perseverative behavior in the WCST may shed light on the interplay between cognitive flexibility and routinization, as affected by repetition, and on the explanatory power of the two neuropsychological theories under consideration (i.e., SAS theory and GIC theory), as illustrated in Figure 2.

There is a growing recognition in psychology that traditional statistical methods, such as null hypothesis significance testing (NHST), may need to be revised. NHST evaluates the significance of observed effects by comparing data to a predetermined threshold (e.g., *α* = 0.05), generating a *p*-value that indicates the likelihood of results under the null hypothesis. However, NHST has been criticized for its rigid thresholds, binary outcomes, potential misinterpretation of *p*-values, and selective reporting (e.g., Meehl, 1978; Szucs and Ioannidis, 2017).

Bayesian statistical methods (Gelman et al., 2013; Gelman and Shalizi, 2013; Kruschke, 2014) have gained popularity in psychology and neuroscience as an alternative approach. Bayesian methods integrate prior knowledge with observed data, allowing for a cumulative evaluation of evidence (Rozeboom, 1960; Benjamin et al., 2018; Wagenmakers et al., 2018a,b; van de Schoot et al., 2021). Bayesian analysis generates probability distributions that provide metrics such as posterior means or credible intervals. In addition, Bayesian hypothesis testing evaluates posterior model probabilities, which helps to directly assess the strength of theories. In this sense, Bayesian statistics is more in line with Popper’s (1934/35) approach to science.

In summary, we have identified three key areas of innovation in neuropsychology: the proliferation of online assessment tools, the turn to theory-driven research, and the shift from frequentist NHST to Bayesian statistics. The present study illustrates how these three innovative trends can be combined to advance neuropsychology. Using the unsupervised online assessment of perseverative behavior on the iWCST, we analyzed the evidence for conflicting predictions about PE derived from two neuropsychological theories, with the goal of rejecting the theory that received less empirical support. As explained above, the manifestation of PE on the iWCST may serve to elucidate the interplay between cognitive flexibility and routinization, as affected by repetition. SAS theory, which focuses on goal-directed control of cognitive processes, predicts more frequently occurring PE in routine situations compared to non-routine situations, i.e., non-repetitive PE (%) < repetitive PE (%). GIC theory, which focuses on retrieval of goal-directed, instrumental memory traces, predicts less frequently occurring PE in repetitive compared to non-repetitive situations, i.e., non-repetitive PE (%) > repetitive PE (%). While the two theories under consideration do not produce numerical point values, they do provide ordinal, informative hypotheses (Hoijtink, 2011). Specifically, H_SAS_, derived from SAS theory, predicts nPE% < rPE%, whereas H_GIC_, derived from GIC theory, predicts nPE% > rPE%. Because the Bayesian statistical framework offers the aforementioned advantages over NHST, we used a Bayesian statistical method called BAIN (BAyesian INformative hypotheses evaluation; Hoijtink et al., 2019) to formally assess the validity of these two theories of the dynamic modulation of cognitive flexibility in light of the observed sample evidence.

The decision to test only GIC versus SAS is based on two considerations. First, SAS is the most established theory and provides a robust benchmark, while GIC is a novel theory developed specifically for this phenomenon. Comparing these two theories allows for a rigorous evaluation of the validity of the GIC theory against the gold standard. Second, by first comparing GIC with SAS and then testing the prevailing theory against others, we envision a thorough evaluation process that integrates insights from multiple theories over time, provided that appropriate diagnostic experimental designs are developed. This strategy of successive pairwise theory tests aims to systematically narrow down to the most accurate theory.

## 2 Materials and methods

Study preparation included the following components: (1) programming the behavioral card-sorting task (iWCST), (2) setting up the online server to host the study, including data storage, and (3) recruiting participants for the study. The schematic workflow of the process of bringing the iWCST online as part of the present study is presented in Supplementary Figure S1.1.

### 2.1 Inviting study participants

Supplementary Figure S1.1 shows that the researchers approached several inviting organizations, such as student offices and student associations, at a number of universities in the southeastern part of the German state of Lower Saxony. The participating universities include the Hannover Medical School (MHH), the Hannover University of Music, Drama and Media (HMTMH), the Hannover University of Veterinary Medicine (TiHo), and the psychology departments of the Technical University of Braunschweig (TU BS) and the University of Hildesheim (U HI).

The collaborating inviting organizations distributed emails prepared by the researchers to their students (see Supplementary Figure S1.2). It contained a hyperlink that connected participants with the server of the online study. Table 1 clearly shows that the invitation emails were more or less successful in terms of the number of participants that could be recruited. Overall, email distribution through official faculty channels (student offices, e.g., MHH) seemed to be much more effective than distribution through more informal channels (student associations, e.g., U HI).

**Table 1:** Number of participants who opened the study hyperlink, number of early and late dropouts, and number of complete and valid records per participating university.

			MHH	HMTMH	TiHo	TU BS	U HI	Total
Opened hyperlink			582	120	20	102	2	826
	*early* dropouts during instructions (no data)		185	53	12	37	2	289
	iWCST started (complete or partial record)		397	67	8	65	0	537
		*late* dropouts - during iWCST	22	5	0	4	0	31
		- during questionnaires	3	0	0	0	0	3
	Complete record		372	62	8	61	0	503
Criteria for inclusion			362	60	8	59	0	489

MHH, Medizinische Hochschule Hannover; HMTMH, Hochschule für Musik, Theater und Medien Hannover; TiHo, Tierärztliche Hochschule Hannover; TU BS, Technische Universität Braunschweig/Institut für Psychologie; U HI, Universität Hildesheim/Institut für Psychologie.

Table 1 shows that a total of *N* = 826 students opened the study hyperlink; we refer to them as neurotypical individuals because they are presumed to have no neurological conditions. Of these 826 individuals, 503 individuals (*N* = 359 females/*N* = 143 males; one preferred not to say) completed the data collection fully (approximately 60.9%). Of the 323 individuals who dropped out, the vast majority (289 individuals, or approximately 89.5 percent of all dropouts) decided not to continue during the task instructions (early dropouts), while only 34 individuals (or approximately 10.5 percent of all dropouts) actually started but did not complete the data collection (late dropouts). Of these 34 late dropouts, 31 did not fully complete the iWCST, while the remaining three late dropouts completed the iWCST fully but did not complete the questionnaires.

Data quality was very good among those who completed the iWCST and questionnaires in full, as only 14 out of 503 records (i.e., no more than approximately 2.8%) had to be excluded based on our pre-specified criteria for iWCST data quality (see below). A total of *N* = 489 participants (*N* = 350 females/*N* = 139 males) provided complete records that fulfilled the inclusion criteria (see below).

The general study instructions are documented in Supplementary Figure S2.1. The basic framing of the study objective was the assessment of “ability to concentrate.” Participants were informed that the task would take approximately half an hour, and that the validity of the study required that they perform the task alone in a quiet environment to allow for undisturbed task performance, without any help of others.

Participants had the opportunity to receive a financial compensation of 10€, which could be claimed by sending an email to the researchers as shown in Supplementary Figure S1.1. A unique 8-digit alpha-numeric code generated by each individual participant for anonymous identification was checked against the records to ensure actual study participation.

### 2.2 Instruments

The online study consisted of the iWCST and a series of demographic questions/psychological questionnaires. These two study components were imported separately into the open source software JATOS 3.8.5 (Lange et al., 2015). Once combined, the MindProbe server^3^ hosted the study and stored data.

JATOS creates several types of hyperlinks. We used the ‘general single’ hyperlink type, which can be distributed to an unlimited number of recipients at once (hence the qualifier ‘general’). However, this general hyperlink can only be used once in the same browser (hence the qualifier ‘single’), thus restricting participants from participating in the study more than once. Each participating university was given a unique ‘general single’ hyperlink, which allowed us to determine the number of participants from each participating university, as shown in Table 1. Details of this study workflow are shown in Supplementary Figure S1.1.

#### 2.2.1 Questionnaires

The demographic questions (age, years of education, and gender) and four questionnaires were embedded in JATOS as HTML files. Table 2 shows the corresponding data. Handedness was assessed using the four-item short form of the Edinburgh Handedness Inventory (EHI-SF; Veale, 2014). The 10-item short form of the Big Five Inventory was used to assess extroversion, conscientiousness, openness, agreeableness, and neuroticism (BFI-10; Rammstedt et al., 2014). Participants also completed the 11-item short form of the Behavioral Inhibition System/Behavioral Approach Inventory (BIS/BAS-11; Studer et al., 2016). Depression and anxiety were assessed using the four-item short form of the Physical Health Questionnaire (PHQ-4; Löwe et al., 2010).

**Table 2:** Descriptive statistics of demographic variables and questionnaire scores.

	*N*	*M*	SD	Mdn	Q1	Q3	Min	Max
Age (in years)	489	23.7	4.4	23.0	21.0	25.0	17.0	68.0
Educational years	488^1^	15.4	2.1	15.0	14.0	17.0	12.0	26.0
Handedness	489	70.3	47.0	87.5	62.5	100.0	−100.0	100.0
BFI-10								
Extroversion	489	6.4	0.9	6.0	6.0	7.0	3.0	9.0
Agreeableness	489	6.5	1.4	6.0	6.0	7.0	3.0	10.0
Conscientiousness	489	6.9	1.2	7.0	6.0	8.0	3.0	10.0
Neuroticism	489	5.9	1.3	6.0	5.0	7.0	2.0	9.0
Openness	489	6.1	1.3	6.0	5.0	7.0	2.0	10.0
BIS/BAS-11								
BIS Total	489	15.8	2.8	16.0	14.0	18.0	5.0	20.0
BAS Total	489	18.0	2.2	18.0	17.0	19.0	11.0	23.0
BAS-FS	489	5.0	1.2	5.0	4.0	6.0	2.0	8.0
BAS-D	489	6.1	1.2	6.0	5.0	7.0	2.0	8.0
BAS-RR	489	6.9	1.0	7.0	6.0	8.0	3.0	8.0
PHQ-4								
PHQ Total	489	3.7	2.6	3.0	2.0	5.0	0.0	12.0
PHQ Depression	489	1.8	1.4	2.0	1.0	2.0	0.0	6.0
PHQ Anxiety	489	1.9	1.6	2.0	1.0	3.0	0.0	6.0

Handedness: Edinburgh Handedness Inventory Short Form-4 items (Veale, 2014). A score of − 100 indicates strong left-handedness, a score of 100 indicates strong right-handedness. “Big Five” Personality Questionnaire BFI-10 (Rammstedt et al., 2014): Big Five Inventory-10 items (scaled from 1 to 5; higher scores indicate stronger item affirmation), factor scores-2 items each (scaled from 2 to 10). Behavioral Inhibition/Activation Scale BIS/BAS-11 items (scaled from 1 to 4, higher scores indicate stronger item affirmation; Studer et al., 2016): Behavioral Inhibition System (BIS)-5 items (scaled from 5 to 20), Behavioral Approach System (BAS)-6 items (scaled from 6 to 24), with BAS-FS (Fun Seeking), BAS-D (Drive), BAS-RR (Reward Responsiveness)-two items each (scaled from 2 to 10). Patient Health Questionnaire PHQ-4 (Löwe et al., 2010): Patient Health Questionnaire-4 items (scaled from 0 to 3, higher scores indicate stronger item affirmation), with PHQ-Total (scaled from 0 to 12) and Depression, Anxiety-two items each (scaled from 0 to 6). ^1^One missing value.

#### 2.2.2 Internet-based Wisconsin card-sorting task

The iWCST was programmed using OpenSesame version 4.0 and OSWeb, which is the JavaScript implementation of OpenSesame (Mathôt and March, 2022). The OpenSesame/OSWeb iWCST program was imported into JATOS 3.8.5 (Lange et al., 2015) installed on the MindProbe server (see text footnote 3, respectively). The authors will provide access to the iWCST program to interested researchers upon reasonable request, preferably by providing additional hyperlinks. These hyperlinks will be sufficient for conducting additional studies using the iWCST in collaboration with the authors.

The iWCST is closely modeled after the M-WCST (Schretlen, 2010), which is an established, commercially available WCST variant. The M-WCST, in turn, shares many features with Nelson’s (1976) MCST variant, such as using only the 24 unambiguous target cards and changing dimensions after only six consecutive correct sorts. Dimensions had to be performed in a fixed order throughout the iWCST, i.e., COLOR, SHAPE, NUMBER, COLOR, SHAPE, etc., in contrast to the standard M-WCST and MCST instructions, but consistent with Heaton’s (1981) and Heaton et al.’s (1993) variant of the WCST.

As shown in Supplementary Figure S1.3, participants match target cards on a trial-by-trial basis to one of the four consistently appearing keycards. All stimuli are presented on a gray background. On each trial, participants encounter four keycards that appear along the horizontal axis at consistent spatial locations on the computer screen. The card-sorting responses can be based on any of the three dimensions of COLOR, SHAPE, and NUMBER of the objects depicted. Each target card selected from the set of 24 unambiguous target cards can be assigned to three *different* keycards based on the COLOR, SHAPE, or NUMBER of the objects depicted; hence the qualifier ‘unambiguous’. On each trial, the selection of the fourth keycard represents an odd error (OE) because it has no common feature with the target card.

The iWCST differs from the manual versions of the WCST in four main ways. First, participants in the iWCST respond by pressing the Y, C, B, or M keys on a QWERTZ keyboard (their spatial arrangement corresponds to Z, C, B, or M on a QWERTY keyboard). This keyboarding requires the use of desktop or laptop computers, excluding the use of touchscreen devices such as tablets or smartphones. Supplementary Figure S1.3 shows how these four letter keys map to the four keycards. To facilitate stimulus–response mapping, the keycards are presented together with their corresponding letter keys.

Second, while manually sorted WCST target cards remain visible for longer periods of time, iWCST target cards disappear after trial-by-trial feedback, as shown in Figure 1 and described below. Each trial begins with the presentation of a target card underneath the keycards, followed by the participant’s response via key press. Upon response, the target card visually moves from its original position to under the selected keycard. Participants then receive feedback indicated by the words “correct” or “incorrect” displayed for 1,200 milliseconds. The target card and feedback then disappear, followed by an 800-millisecond intertrial interval before the next trial begins. These relatively short temporary appearances, which are unique to the iWCST, place a relatively higher demand on working memory than traditional manual versions of the WCST (Lange et al., 2016).

A third major departure from manual card-sorting tasks concerns the trial-by-trial sequence of target cards. While manual WCST versions typically follow a predetermined order, the iWCST uses a pseudo-random selection of target cards from the set of 24 different target cards. This pseudo-random order ensures that the same target card never appears on two consecutive trials. The pseudo-random order also maintains a 50% chance of encountering non-repetitive versus repetitive opportunities to commit PE. This guarantees an equal chance of encountering trials in which the action-relevant stimulus features are repeated or not repeated. Thus, the pseudo-random sequence provides an equal opportunity for non-repetitive and repetitive PE occurrences. Note that the distinction between non-repetitive and repetitive PE excludes exact repetitions of target cards on successive trials and focuses on the alternation or repetition of current action-relevant features on trials following informative error feedback as shown in Figure 1.

Fourth, the iWCST is adapted to optimize the assessment of PE, which is a primary rationale for the study. The termination condition in the iWCST sets a maximum of 240 trials, but allows for early termination if the participant encounters 20 trials each of potential non-repetitive and repetitive PE before reaching this limit. This approach strikes a balance between obtaining adequate data for reliable cognitive assessment (Kopp et al., 2021) and preventing undue prolongation of the test or participant fatigue. The iWCST’s flexibility in stopping the assessment optimizes duration while ensuring sufficient data collection.

Table 3 shows the resulting number of trials and the number of trials for potential non-repetitive and repetitive PE. Inspection of Table 3 shows that the average number of trials is more than 200 trials, and that the average number of opportunities to perform non-repetitive and repetitive PE is slightly more than 20 trials each. The latter aspect of the iWCST is important because it allows the main measures, non-repetitive PE (%) and repetitive PE (%), to be assessed with comparable psychometric quality. To date, it has not been possible to achieve psychometrically matched measures of non-repetitive and repetitive PE with manual card-sorting tasks such as the M-WCST (Schretlen, 2010), due to the inherent imbalance between opportunities for non-repetitive and repetitive PE (Kopp et al., 2019).

**Table 3:** Descriptive sample statistics of the iWCST variables.

	*N*	*M*	SD	Mdn	Q1	Q3	Min	Max
N_total_trials	489	206.3	31.5	209.0	184.0	240.0	90.0	240.0
N_categories	489	24.0	5.4	24.0	20.0	28.0	7.0	34.0
categories%^1^	489	11.5	1.3	11.7	10.8	12.5	6.8	14.2
PE numbers								
N_occ_PE	489	42. 9	4.9	43.0	41.0	46.0	18.0	63.0
N_PE	489	2.9	2.4	2.0	1.0	4.0	0.0	14.0
N_occ_nPE	489	21.5	4.0	20.0	20.0	23.0	10.0	43.0
N_nPE	489	2.0	1.9	2.0	1.0	3.0	0.0	11.0
N_occ_rPE	489	21.4	4.0	20.0	20.0	23.0	8.0	35.0
N_rPE	489	0.9	1.2	1.0	0.0	1.0	0.0	8.0
PE percentages								
nPE%^2^	489	9.2	8.2	8.0	4.0	13.6	0.0	45.0
rPE%^3^	489	3.9	5.3	3.6	0.0	5.0	0.0	31.3
ESE^4^	489	5.3	8.7	4.74	0.0	10.0	−23.1	40.0
ESE/sum^5^	430^6^	0.4	0.6	0.55	0.0	1.0	−1.0	1.0
Other error types								
N_occ_SLE	489	137.3	27.2	140.0	117.0	161.0	41.0	187.0
N_SLE	489	6.6	3.9	6.0	4.0	9.0	0.0	24.0
SLE%^7^	489	5.1	3.4	4.6	2.6	6.8	0.0	19.6
N_occ_IE	489	11.2	4.5	12.0	8.0	14.0	0.0	24.0
N_IE	489	1.9	2.0	1.0	0.0	3.0	0.0	10.0
IE%^8^	487^9^	14.9	13.8	12.5	0.0	25.0	0.0	66.7
N_OE	489	0.9	1.5	0.0	0.0	1.0	0.0	11.0

N, number of …; occ, occasion; PE, perseveration errors; nPE, non-repetitive PE; rPE, repetitive PE; ESE, error suppression effect; SLE, set-loss errors; IE, integration errors; OE, odd errors. ^1^Percentage of N_categories on N_total_trials. ^2^Percentage of N_nPE on N_occ_nPE; ^3^Percentage of N_rPE on N_occ_rPE; ^4^nPE% - rPE%; ^5^(nPE% - rPE%)/(nPE% + rPE%); ^6^59 missings because in these cases the sum of nPE% + rPE% equaled 0; ^7^Percentage of N_SLE on N_occ_SLE; ^8^Percentage of N_IE on N_occ_IE; ^9^Two missings because in these cases N_occ_IE was 0.

Participants receive detailed task instructions prior to the start of the iWCST. The formulation of appropriate task instructions is very important because there is no other way to comprehensively instruct the card-sorting task in the context of an unsupervised online assessment. Supplementary Figures S2.2–S2.23 provide the complete iWCST instructions, which include a general description of the sorting task at hand, interactive elements to ensure that participants have a good understanding of the basics of the card-sorting task, and information about occasional dimension changes. Supplementary Figures S2.1–S2.22 provide detailed information on how these dimension changes were instructed in the iWCST. Note that the iWCST instructions never explicitly mention the three dimensions underlying the sorting of the cards, i.e., the COLOR, SHAPE, and NUMBER of objects depicted, leaving room for cognitive processes involved in categorizing information. Thus, the iWCST requires abstraction and concept formation, much like the original WCST. Abstraction involves extracting general properties from sensory input, while conceptualization goes beyond direct sensory experience and involves the formation of abstract concepts for higher-level organization of information.

The iWCST is administered immediately following the completion of the task instructions. The average time to complete the iWCST and questionnaires is less than 30 min (*Mdn* = 22.65 min, *IQR* = 7.15 min).

#### 2.2.3 Internet-based Wisconsin card-sorting task measures

As shown in Table 3, the iWCST measures include the number of trials completed, the number of categories achieved, where a category is defined as six consecutive correct sorts, and the percentage of categories among the number of completed trials. PE are the main measures of the iWCST, notably non-repetitive PE (%) and repetitive PE (%). Therefore, the iWCST records the number of occasions for committing PE, non-repetitive PE, and repetitive PE, as well as the actual number of PE, non-repetitive PE, and repetitive PE, as defined in Figure 1, allowing the percentage of PE, non-repetitive PE, and repetitive PE to be calculated.

**Figure 3:**
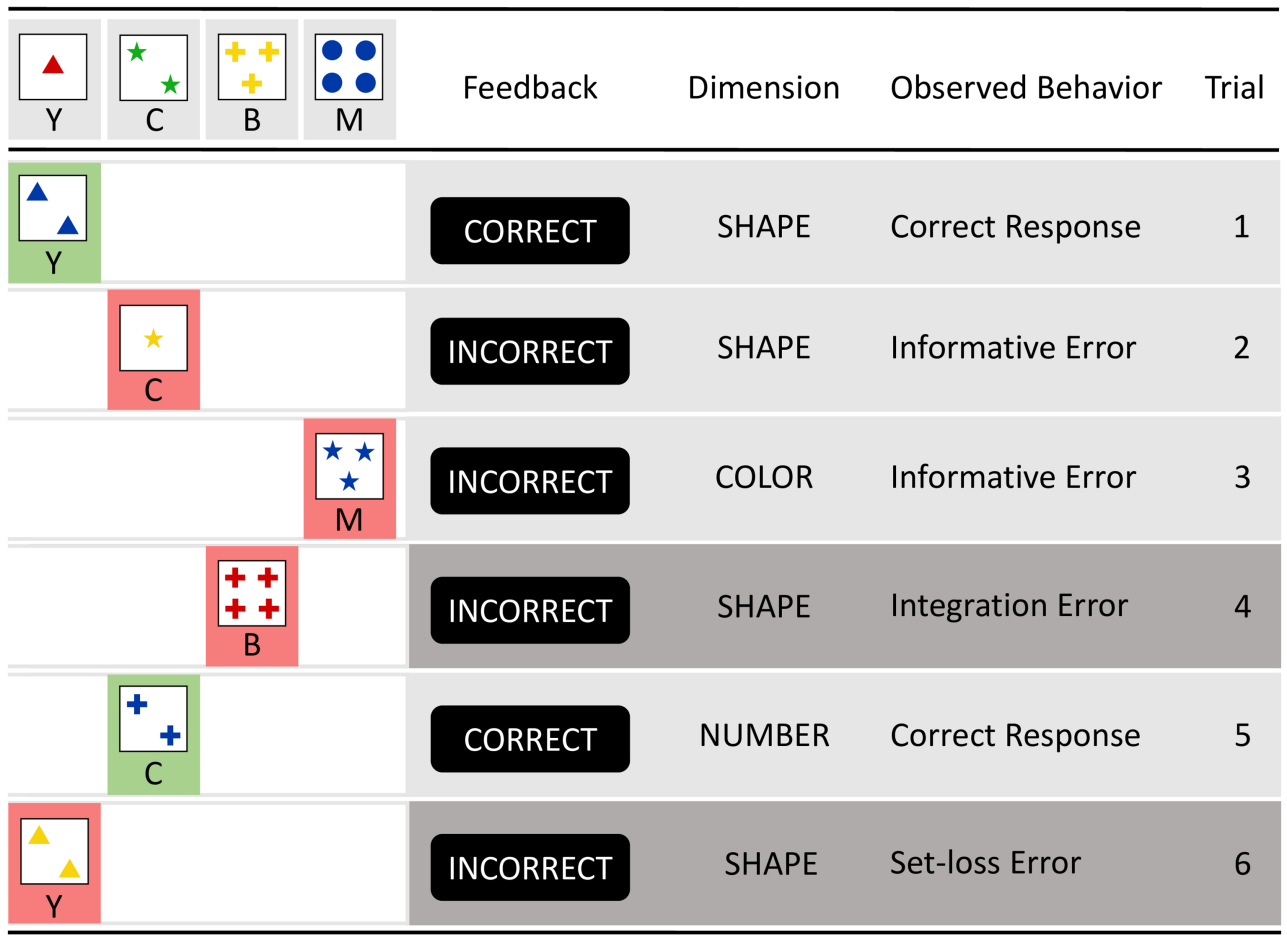
This figure illustrates the typical layout of computerized Wisconsin card-sorting tasks and two additional types of errors, i.e., integration errors and set-loss errors. Each row represents a new trial on which participants must match a trial-specific target card with one of the four consistently appearing keycards. After applying the dimension SHAPE on the first trial and receiving the CORRECT feedback, the sorting by the dimension SHAPE is repeated on trial 2. However, the INCORRECT informative error signal indicates that the relevant dimension has changed. Trial 3 shows the application of the dimension COLOR, but the INCORRECT feedback indicates that COLOR is also currently incorrect. Trial 4 shows the application of the dimension SHAPE, even though informative error signals have already been issued regarding SHAPE (on trial 2) and COLOR (on trial 3), illustrating an integration error (after excluding SHAPE and COLOR, NUMBER remains the only viable dimension). Trial 5 shows the application of the dimension NUMBER, and the CORRECT feedback indicates that NUMBER is indeed the current correct dimension. However, trial 6, which shows the application of the dimension SHAPE, illustrates a set-loss error because the dimension NUMBER was correctly applied on the previous trial.

Figure 3 shows two additional types of errors, namely integration errors (Lange et al., 2016) and set-loss errors (Kopp, in press). An integration error (IE) occurs after receiving informative error feedbacks on two consecutive iWCST trials that inform about the current incorrectness of two dimensions (such as the second and third trials in Figure 3). Successful integration of the previous two informative error feedbacks results in the selection of the remaining viable dimension, whereas faulty integration of the previous two informative error feedbacks results in the repetition of the dimension excluded by the first informative error feedback. In the example in Figure 3, sorting by SHAPE on Trial 4 is an IE because the SHAPE dimension was already eliminated by the informative error feedback on Trial 2. Successful integration of the previous two informative error feedbacks would have eliminated both the SHAPE and COLOR dimensions, resulting in sorting by NUMBER on Trial 4. An example of successful integration is shown on Trial 5, where sorting by NUMBER correctly follows from integrating the previous two informative error feedbacks, which eliminated both the SHAPE and COLOR dimensions.

Set-loss errors (SLE) are comparatively simple types of iWCST errors, as shown on Trial 6 in Figure 3. SLE occur when participants fail to maintain a cognitive set on an iWCST trial despite receiving feedback that sorting according to that set was correct on the previous trial. For both types of errors, IE and SLE, we calculated the number of occasions for them, as well as their actual number, which in turn allowed us to calculate the percentage of IE and SLE (Table 3). Finally, the number of OE was calculated.

### 2.3 Data analysis

Data preprocessing was performed in Microsoft Excel 2016 (Microsoft Corporation, 2016). Participants with complete records were excluded if they scored more than three standard deviations (a) below the average number of categories achieved, or (b) above the average number of OE (see also Steinke et al., 2020a). In both cases, the presence of these unrepresentative measures may indicate insufficient effort on the task. The application of this criterion resulted in the exclusion of 12 participants. In addition, two participants were excluded because, contrary to our intent, they had fewer than eight occasions of either non-repetitive or repetitive PE. Inspection of Table 1 shows that only 14 out of 503 records had to be excluded based on these criteria, leaving a sample of *N* = 489 valid records.

Bayesian data analysis was performed using JASP version 0.18.3.^4^ Confirmatory data analyses included Bayesian paired samples *t*-tests (Faulkenberry et al., 2020), which provided informal evidence regarding the fit of SAS theory and GIC theory. We finally conducted Bayesian informative hypotheses evaluation via BAIN paired-samples *t*-tests, implemented in the JASP-BAIN module (Hoijtink et al., 2019), to calculate posterior model probabilities of hypothesis H_SAS_, derived from SAS theory and predicting nPE% < rPE%, and of hypothesis H_GIC_, derived from GIC theory and predicting nPE% > rPE%.

Exploratory data analyses included Bayesian correlation and network (Huth et al., 2023) analyses to examine relationships between the iWCST measures. Bayesian regression analyses (van den Bergh et al., 2021) examined relationships between the iWCST measures (as dependent variables) and questionnaire scores (as covariates).

## 3 Results

### 3.1 Descriptive iWCST results

Table 3 shows the descriptive sample statistics for all of the iWCST variables for the participants who were included in the final analysis (*N* = 489). Participants completed a mean of 206.3 iWCST trials, ranging from 90 to 240 trials. Participants achieved a mean of 24.0 iWCST categories, ranging from 7 to 34 categories.

For iWCST error scores, the description of the sample statistics for PE is deferred to the confirmatory analyses. Participants made an average of 6.6 iWCST SLE, ranging from 0 to 24 SLE. The number of SLE occasions averaged 137.3 iWCST trials, ranging from 41 to 187 trials. This resulted in an average of 5.1 SLE%, ranging from 0 to 19.6 percent. Participants made an average of 1.9 iWCST IE, ranging from 0 to 10 IE. The number of IE occasions averaged 11.2 iWCST trials, ranging from 0 to 24 trials. This resulted in an average of 14.9 IE%, ranging from 0 to 66.7 percent. Participants made an average of 0.9 iWCST OE, ranging from 0 to 11 OE.

### 3.2 Confirmatory Bayesian data analysis

**Figure 4:**
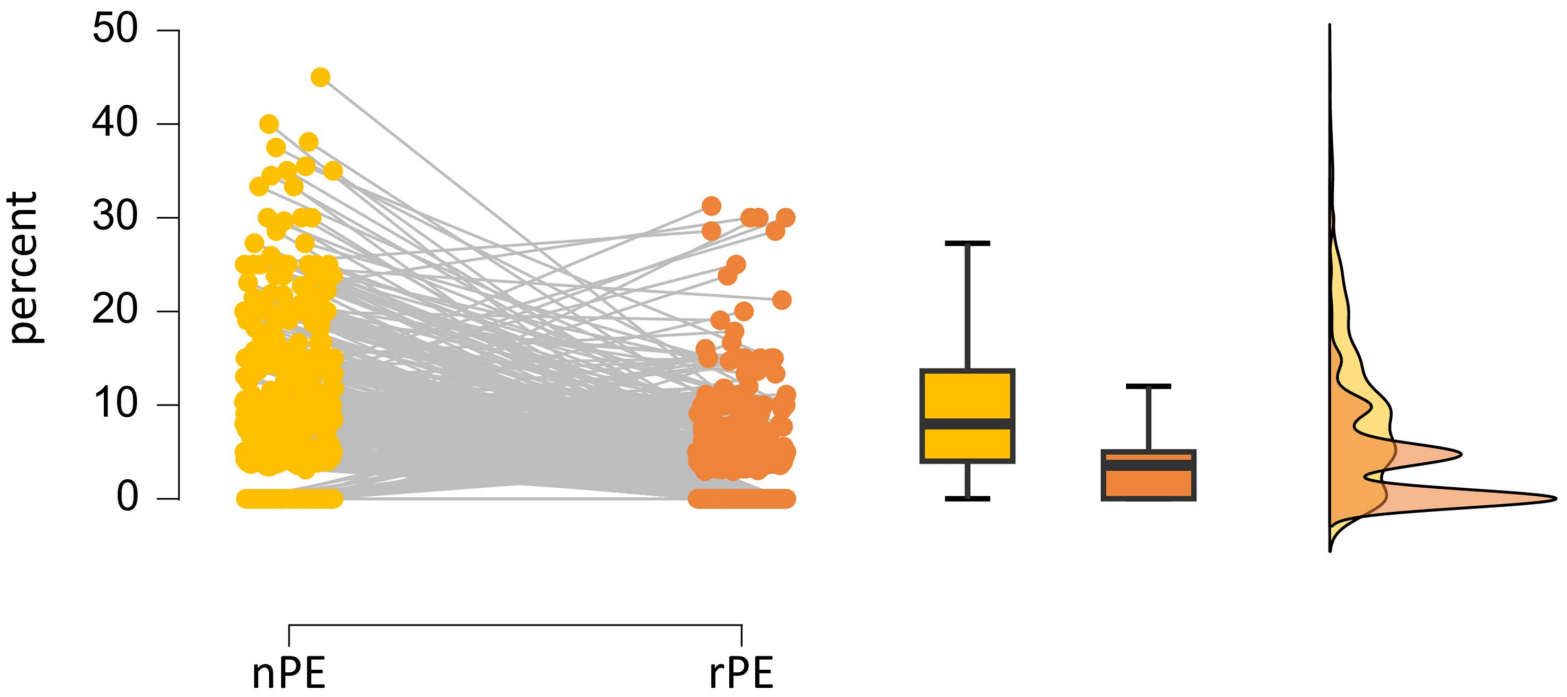
Individual percentages of non-repetitive PE (nPE; in yellow) and repetitive PE (rPE; in orange) are shown on the left. Box and raincloud plots of sample statistics of nPE% and rPE% are shown on the right.

Table 3 shows that participants made an average of 2.9 iWCST PE, ranging from 0 to 14 PE. The number of PE occasions averaged 42.9 iWCST trials, ranging from 18 to 63 trials. Regarding the theoretical distinction between non-repetitive and repetitive PE, the sample statistics are that participants committed an average of 2.0 iWCST nPE, ranging from 0 to 11 nPE. The number of nPE occasions averaged 21.5 iWCST trials, ranging from 10 to 43 trials. This resulted in an average of 9.2 nPE%, ranging from 0 to 45.0 percent. Participants committed an average of 0.9 iWCST rPE, ranging from 0 to 8 rPE. The number of rPE occasions averaged 21.4 iWCST trials, ranging from 8 to 35 trials. This resulted in an average of 3.9 rPE%, ranging from 0 to 31.3 percent. The iWCST PE sample data, the main target of the present study, are also visualized separately for nPE and rPE in Figure 4.

If *δ* is the standardized difference between nPE% and rPE%, then *δ* provides a measure of the effect size, indicating how large the difference between the two error percentages is relative to the variability within the data. Both theories under consideration predict that nPE% and rPE% will be different in the neurotypical population, with the SAS theory predicting *δ* < 0 and the GIC theory predicting *δ* > 0. Note that the number of opportunities to commit either nPE or rPE was nearly identical, as discussed in detail above, with nPE opportunities averaging 21.5 iWCST trials and rPE opportunities averaging 21.4 iWCST trials (see Table 3). Importantly, therefore, the possible existence of a population difference cannot be attributed to the confounding factor that there was a difference in the number of iWCST trials in which non-repetitive PE and repetitive PE were possible.

**Figure 5:**
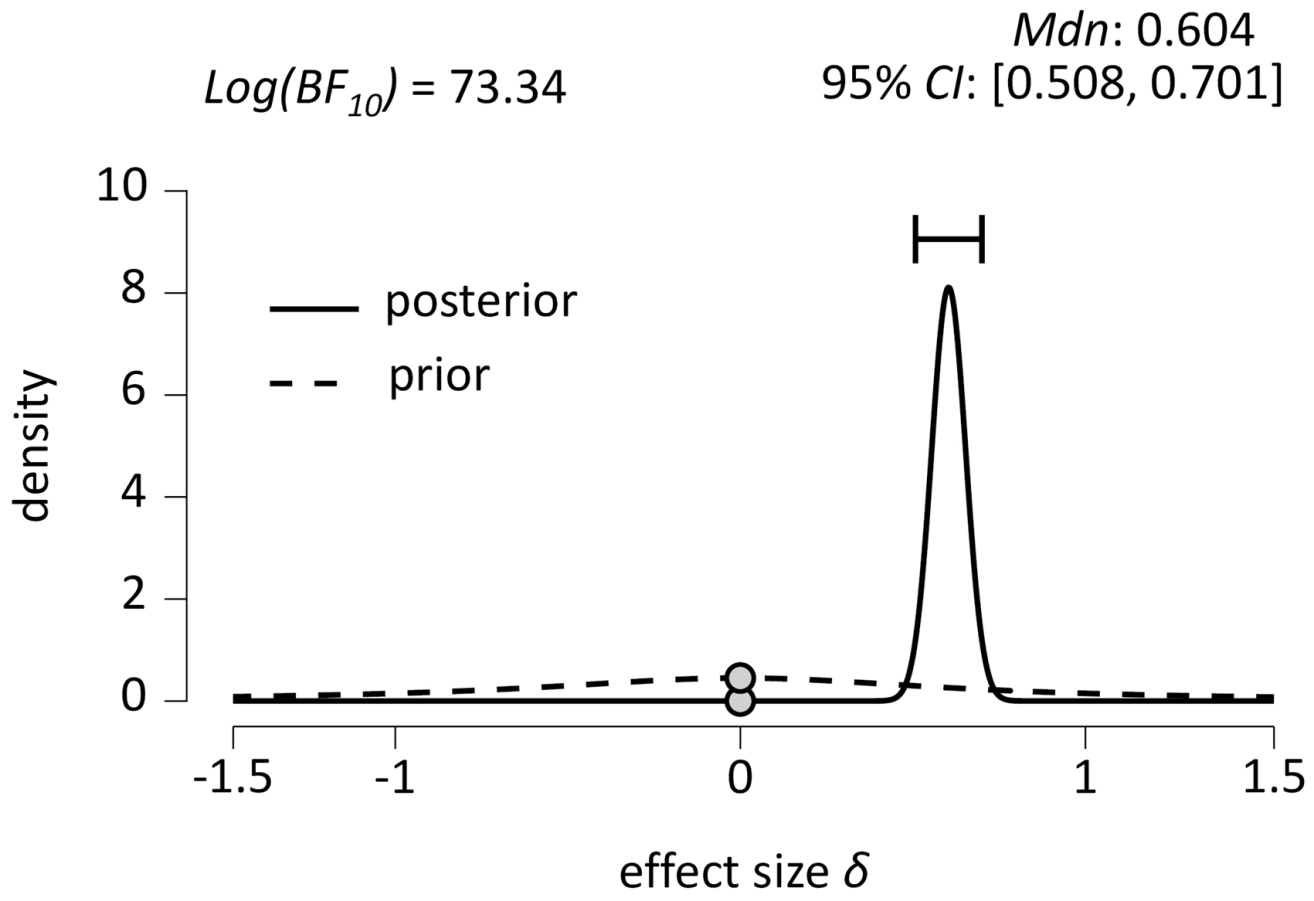
Inferential plot depicting the (default) prior distribution and the obtained posterior distribution (*N* = 489). 95% CI = 95 percent credible interval.

The inferential plot, including the default prior distribution and the obtained posterior distribution, is shown in Figure 5. We used a Bayesian paired samples *t*-test to test the credibility of two models of nPE% and rPE% in the neurotypical population (H0 (*δ* = 0) vs. H1 (*δ* ≠ 0)). For a two-tailed test of H0 (*δ* = 0) vs. H1 (*δ* ≠ 0), we used JASP’s default settings, i.e., a two-parameter Cauchy *prior* distribution (with (central) location *δ* = 0, width *r* = 1/√2 = 0.707), as shown by the dashed line in the graph in Figure 5. The solid line in the graph in Figure 5 shows the obtained *posterior* distribution.

We ran the test to index support for H1 over H0 and found a log Bayes factor *Log(BF_10_)* = 73.34, indicating decisive (Jeffreys, 1939) evidence in favor of H1 (*δ* ≠ 0). Note that the y-coordinate at *δ* = 0 in the posterior distribution is smaller than that of the prior distribution at *δ* = 0. This corresponds to the fact that our posterior belief in H0 (*δ* = 0) has decreased after observing the data. In fact, it has decreased by the obtained log Bayes factor of *Log(BF_10_)* = 73.34. This log Bayes factor can be represented as the ratio of the prior to the posterior on the population effect *δ* under H0 (Faulkenberry et al., 2020).

Figure 5 also shows estimates for the effect size parameter *δ*, with a median effect size of *δ* = 0.604, indicating that the more probable population values of *δ* are centered around *δ* = 0.604, and a 95% credible interval between 0.508 and 0.701, providing a measure of the uncertainty in the size of the population effect *δ* after observing the data. In other words, under the assumption that H0 is false (i.e., that *δ* ≠ 0), there is a 95% probability that the true value of the population effect *δ* falls between 0.508 and 0.701 (Faulkenberry et al., 2020). This inference is clearly inconsistent with the prediction of SAS theory (*δ* < 0), but it is consistent with the prediction of GIC theory (*δ* > 0).

**Figure 6:**
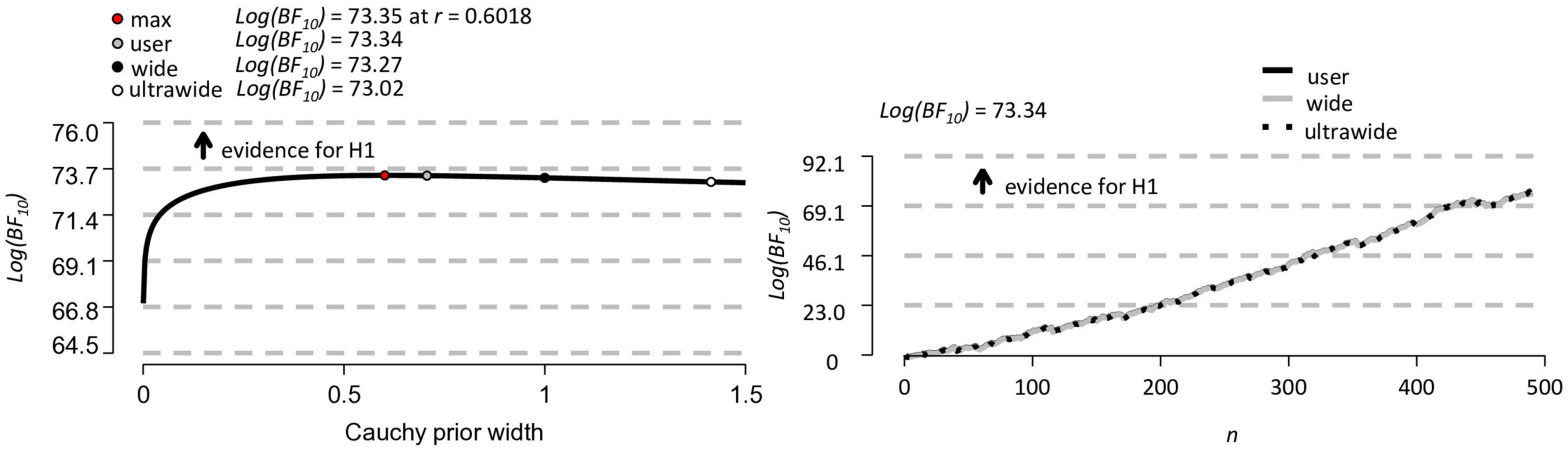
Bayesian robustness check (on the left) and Bayesian sequential analysis (on the right) for the *N* = 489 participants.

The left panel of Figure 6 shows a Bayesian robustness check. The plot shows that the log Bayes factor is robust to changes in the width parameter *r* of the Cauchy prior distribution, with all results *Log(BF_10_)* ≥ 73.02. The invariance of the log Bayes factor to variations in the width of the Cauchy prior (max log Bayes factor, user-defined, wide and ultrawide width) indicates that the results are robust in that they do not depend in an important way on our particular user-defined *r* = 0.707. The right panel of Figure 6 shows a Bayesian sequential analysis. The plot shows increases in the log Bayes factor as data from more and more participants are added under the user-defined, wide, and ultra-wide widths of the Cauchy prior.

**Figure 7:**
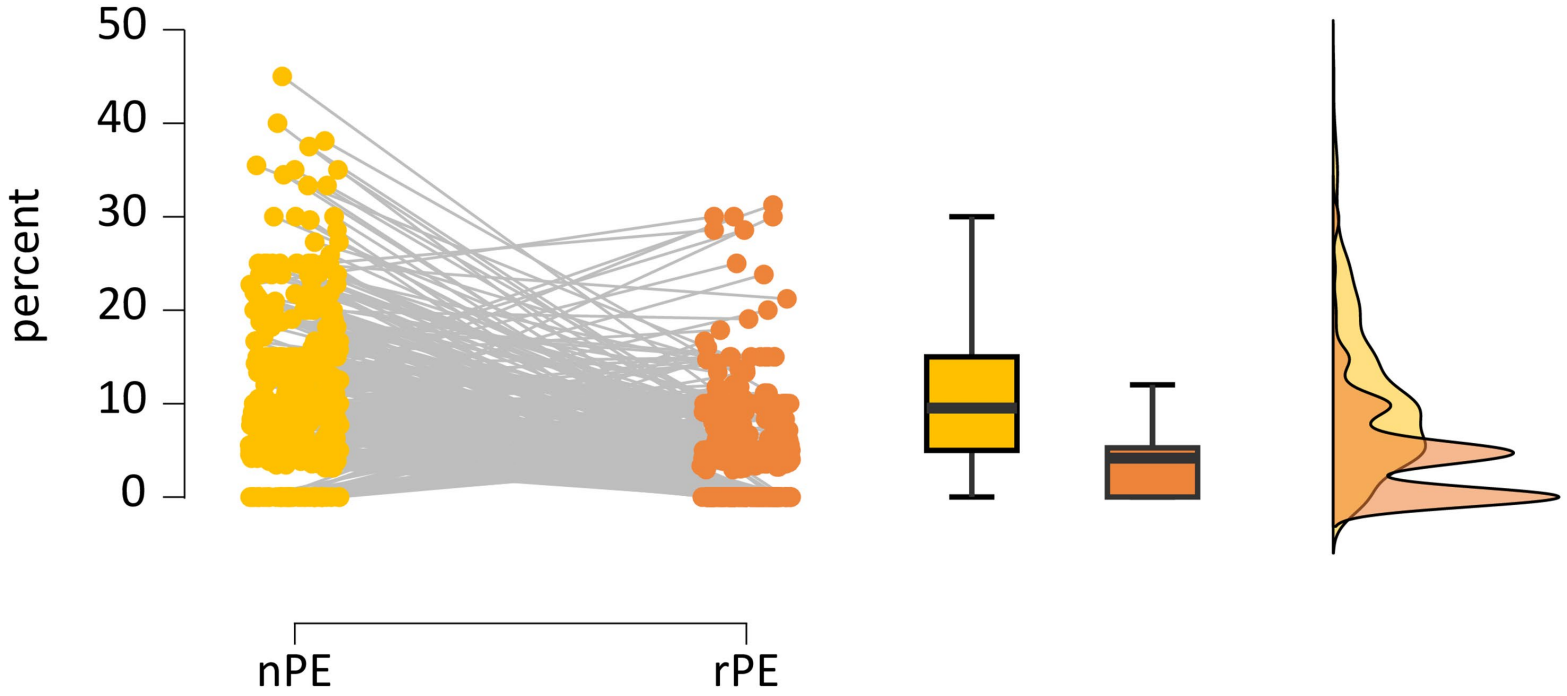
Individual percentages of non-repetitive PE (nPE; in yellow) and repetitive PE (rPE; in orange) are shown on the left after exclusion of *N* = 59 PE-free individuals, leaving *N* = 430 participants. Box and raincloud plots of sample statistics of nPE% and rPE% are shown on the right.

After excluding N = 59 participants who did not perform any PE, N = 430 participants who performed one or more PE remained for analysis. In this subsample, an average of 3.2 iWCST PE was found, ranging from 1 to 14 PE. The number of PE occasions averaged 43.2 iWCST trials, ranging from 27 to 63 trials. Regarding non-repetitive PE and repetitive PE, participants committed an average of 2.3 iWCST nPE, ranging from 0 to 11 nPE. The number of nPE occasions averaged 21.6 iWCST trials, ranging from 10 to 43 trials. This resulted in an average of 10.5 nPE%, ranging from 0 to 45.0 percent. Participants committed an average of 1.0 iWCST rPE, ranging from 0 to 8 rPE. The number of rPE occasions averaged 21.6 iWCST trials, ranging from 12 to 35 trials. This resulted in an average of 4.5 rPE%, ranging from 0 to 31.3 percent. The iWCST PE sample data for N = 430 are visualized separately for nPE and rPE in Figure 7.

**Figure 8:**
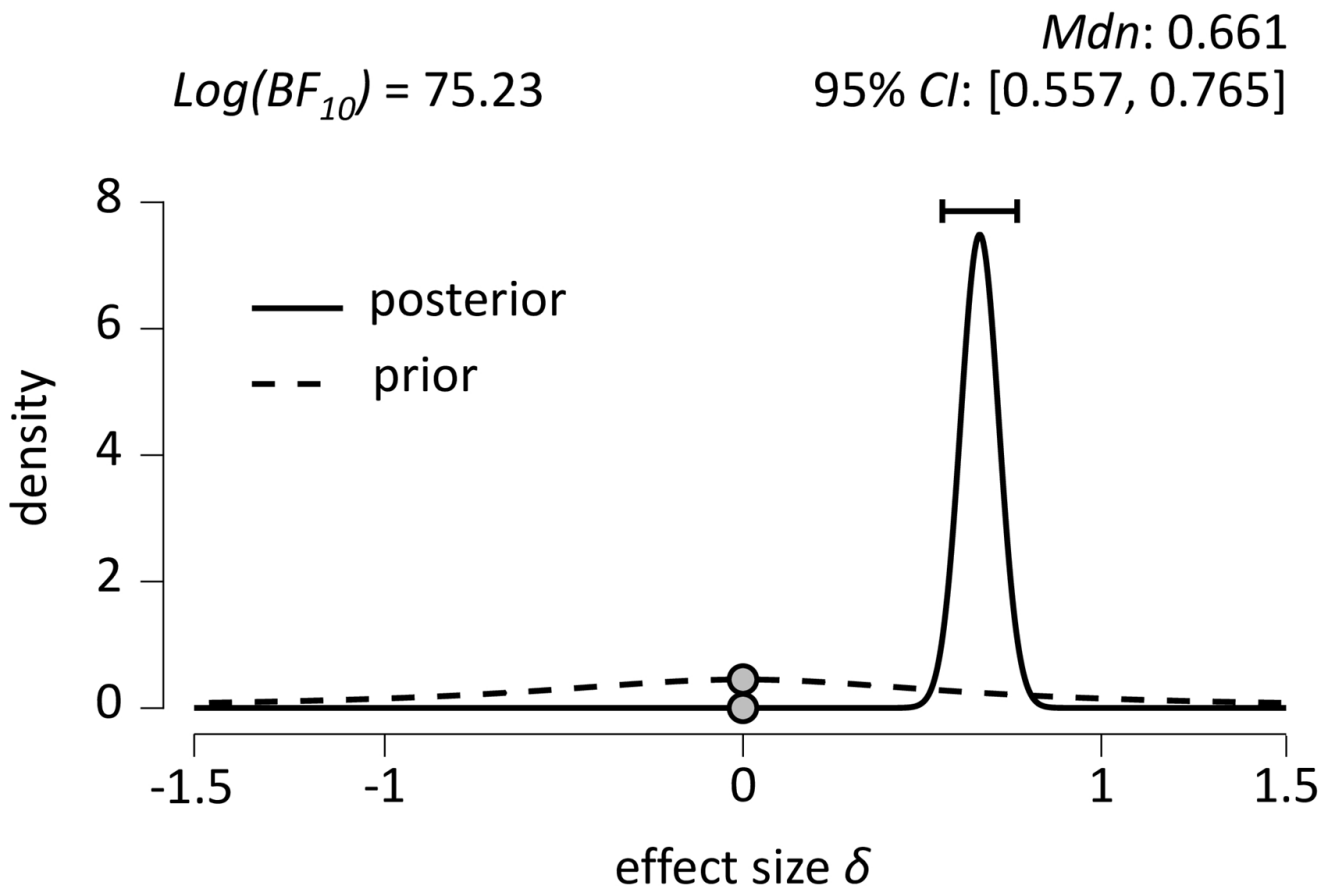
Inferential plot depicting the (default) prior distribution and the obtained posterior distribution (*N* = 430). 95% CI = 95 percent credible interval.

The inferential plot, including the default prior distribution and the obtained posterior distribution, is shown in Figure 8. We again used a Bayesian paired samples *t*-test to test the credibility of the two models of nPE% and rPE% in *N* = 430 participants. The Cauchy *prior* distribution is shown again by the dashed line in the graph in Figure 5, while the solid line shows the obtained *posterior* distribution.

We ran the test to index support for H1 over H0 and found a log Bayes factor *Log(BF_10_)* = 75.23, again indicating decisive (Jeffreys, 1939) evidence in favor of H1 (*δ* ≠ 0). Figure 5 also shows that the median effect size is *δ* = 0.661, with a 95% credible interval between 0.557 and 0.765. Again, assuming that H0 is false (i.e., that *δ* ≠ 0), there is a 95% probability that the true value of the population effect *δ* falls between 0.557 and 0.765 (Faulkenberry et al., 2020), which is again inconsistent with what SAS theory predicts (*δ* < 0), but consistent with what GIC theory predicts (*δ* > 0).

**Figure 9:**
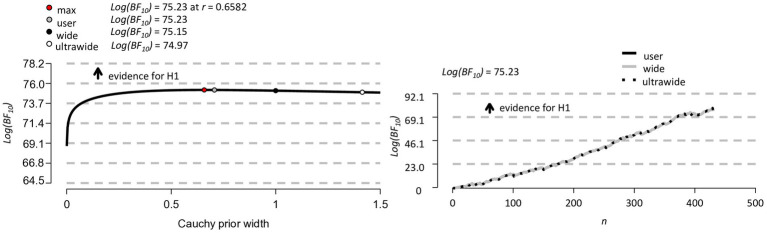
Bayesian robustness check (on the left) and Bayesian sequential analysis (on the right) for *N* = 430 participants.

The Bayesian robustness check on left panel of Figure 9 shows that the log Bayes factor is again robust to changes of the width of the Cauchy prior, with all results *Log(BF_10_)* ≥ 74.97. The right panel of Figure 6 shows a Bayesian sequential analysis, showing increases in the log Bayes factor as data from more and more participants are added under the user-defined, wide, and ultra-wide widths of the Cauchy prior.

Taken together, the results of these confirmatory Bayesian data analyses are remarkably clear. First, we obtained conclusive evidence against H0 (*δ* = 0) that there is no difference between non-repetitive and repetitive PE percentages in the neurotypical population. This conclusion could be drawn both from the analysis of the full sample and from the subsample of participants who committed one or more PE. Second, assuming that H0 is false (i.e., that *δ* ≠ 0), the 95% probability that the true value of the population effect *δ* falls between 0.508 and 0.701 (the central tendency equals 0.604 based on the full sample) or 0.557 and 0.765 (the central tendency equals 0.661 based on the subsample). In both cases, the results are inconsistent with what SAS theory predicts (*δ* < 0), but consistent with what GIC theory predicts (*δ* > 0).

The formal assessment of these two theories was accomplished by BAIN Bayesian paired-samples *t*-tests (Hoijtink et al., 2019). BAIN analyses allow direct comparison of the posterior model probabilities of H_SAS_, derived from SAS theory and predicting nPE% < rPE%, and of H_GIC_, derived from GIC theory and predicting nPE% > rPE%. Based on the full sample (*N* = 489), and assuming equal prior model probabilities, the log Bayes factor in favor of the GIC theory was *Log(BF_GIC-SAS_)* = 93.65, corresponding to posterior model probabilities of *p* > 0.999 for the GIC theory and *p* < 0.001 for the SAS theory. Based on the restricted sample (*N* = 430), again assuming equal prior model probabilities, the log Bayes factor in favor of the GIC theory was *Log(BF_GIC-SAS_)* = 98.47, corresponding to posterior model probabilities of *p* > 0.999 for the GIC theory and *p* < 0.001 for the SAS theory. Therefore, based on the observed data, the GIC theory is probably (even almost certainly) correct in predicting the ESE, while the SAS theory is probably (even almost certainly) incorrect in describing cognitive processes involved in the dynamics of cognitive flexibility.

### 3.3 Exploratory Bayesian data analysis

We performed a Bayesian correlation analysis to examine the associations between iWCST measures, namely categories%, SLE%, nPE%, rPE%, and IE%. As expected, categories%, with higher values indicating better performance, and error variables, with lower values indicating better performance, are negatively correlated (Table 4). Categories% and SLE% show the strongest degree of association. The first-order correlations between error variables are positive but moderate at best, ranging from *r* = 0.35 (SLE% and nPE%) to *r* = 0.15 (IE% and rPE%).

**Table 4:** Results of the Bayesian correlation analysis.

Variable		Categories%	SLE%	nPE%	rPE%	IE%
SLE%	Pearson’s *r*	**−0.91**				
	*Log(BF₁₀)*	411.54				
nPE%	Pearson’s *r*	**−0.49**	**0.35**			
	*Log(BF₁₀)*	62.22	27.88			
rPE%	Pearson’s *r*	**−0.26**	**0.18**	**0.22**		
	*Log(BF₁₀)*	14.45	4.99	9.12		
IE%	Pearson’s *r*	**−0.39**	**0.28**	**0.18**	**0.15**	
	*Log(BF₁₀)*	36.56	16.59	4.71	2.31	

Categories%, percentage of categories; SLE%, percentage of set-loss errors; nPE%, percentage of non-repetitive perseveration errors; rPE%, percentage of repetitive perseveration errors; IE%, percentage of integration errors. All *N* = 489; except correlations involving IE%, *N* = 487. correlation co-efficients in bold.

We also performed a Bayesian network analysis on these cross-sectional iWCST multivariate data (Borsboom et al., 2021) using JASP’s Network module (Huth et al., 2023). To do this, we have used the default settings with the following exceptions: The GCGM (Gaussian copula graphical model, mixed variables) estimator was preferred over the GCM (Gaussian graphical model, continuous variables) estimator, and the iteration and burn-in sampling parameters were increased to 50,000 (instead of 10,000) and 20,000 (instead of 5,000), respectively. Finally, the prior edge inclusion probability was set to 0.10 (instead of 0.50).

**Figure 10:**
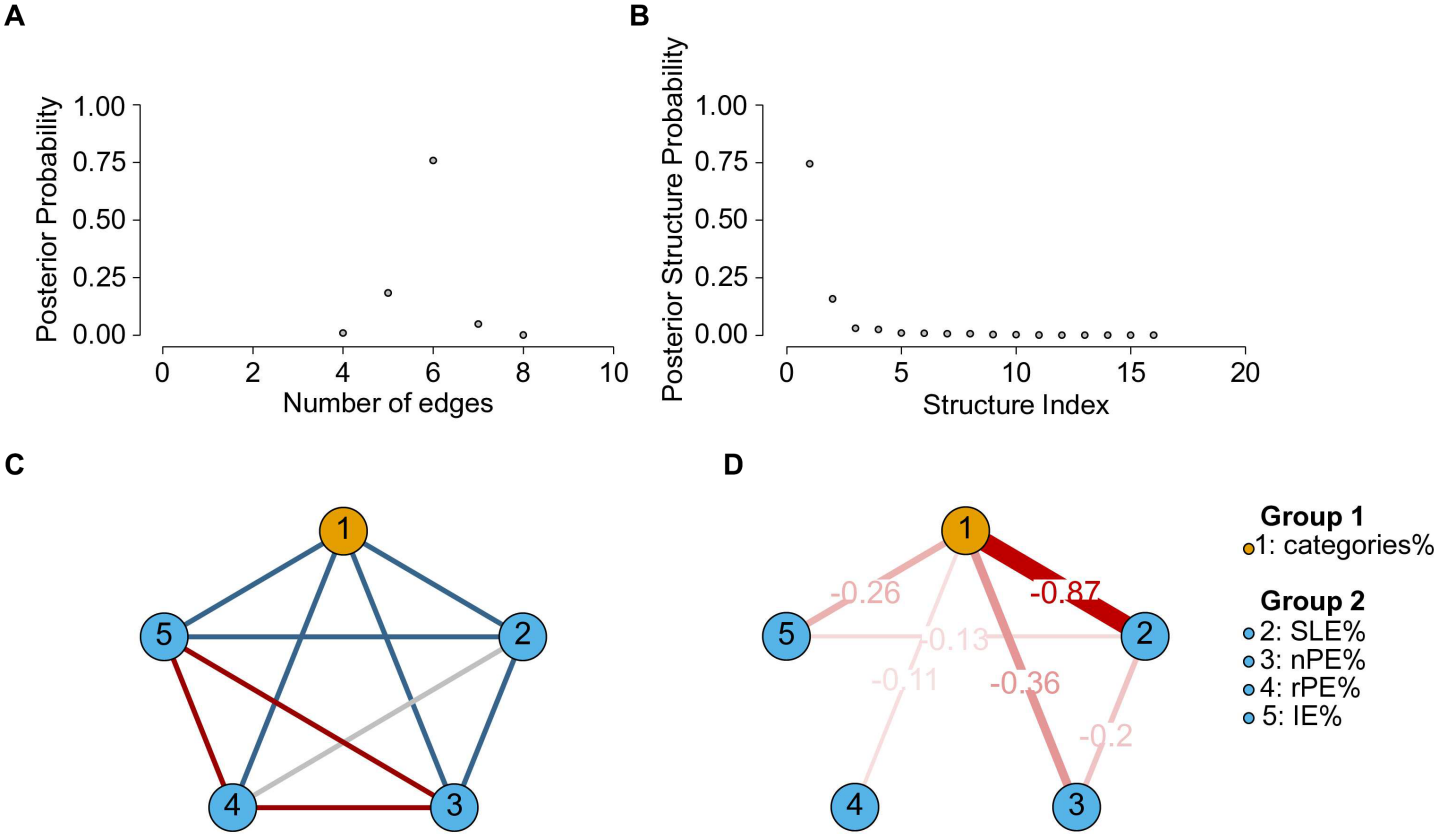
Bayesian network analysis results of categories%, SLE%, nPE%, rPE%, and IE% iWCST measures for *N* = 489. **(A,B)** Are concerned with network structure uncertainty and show the posterior probabilities of different network structures and their complexity. **(A)** Shows the posterior probability of different structural complexities, where the complexity is the network density. The peak posterior probability occurs at a density of six edges. **(B)** Shows the posterior probabilities of the visited structures, sorted from most to least probable. A single structure clearly stands out as the most probable, with a posterior probability around 0.75. **(C,D)** Are concerned with edge inclusion and exclusion and show details of the most probable network structure. **(C)** Edge evidence plot showing six edges present in blue (evidence for inclusion, *BF_10_* > 10), three edges absent in red (evidence for exclusion, *BF_10_* < 0.10), and one edge with insufficient evidence (0.10 < *BF_10_* < 10) in gray. **(D)** The corresponding parameter estimate plot shows edges with *BF_10_* > 10 and their model-averaged parameter estimates. Edge thickness and saturation represent the strength of the association; only negative relationships are present (shown in red). Note that an analysis of the uncertainty in the precision of the edge parameter estimates is not possible with the JASP Network module.

Figure 10 shows the results of this Bayesian network analysis, which is based on partial correlations between the variables. As can be seen, the resulting network structure retains moderate complexity (including six edges), with one single structure assembling a posterior probability of about 0.75. The most likely network structure includes all edges between categories% and the error variables, SLE%, nPE%, rPE%, and IE%, with parameter estimates ranging from −0.87 (edge connecting categories% to SLE%) to −0.11 (edge connecting categories% to rPE%), plus two edges connecting error variables (edges connecting SLE% to nPE% and SLE% to IE%, respectively).

Overall, categories% appears as a strongly connected node (four edges), while rPE% appears as a weakly connected node (only one edge, which is also the weakest edge included). These impressions are clearly supported by the centrality measures (Supplementary Table S3.1), which show that categories% is the most central node in terms of betweenness, closeness, and strength, while rPE% is the least central node in terms of betweenness, closeness, and strength.

In addition to these analyses of internal iWCST associations, we analyzed associations between iWCST variables and the questionnaires we used (see Table 2; BFI-10, Rammstedt et al., 2014; BIS/BAS-11, Studer et al., 2016; PHQ-4, Löwe et al., 2010). The results of these Bayesian regression analyses can be found in the Supplementary Table S3.2. A notable finding reported there is that none of the questionnaire scores contributed significantly to the prediction of the iWCST measures, including PE variables. That is, neither personality variables, as indexed by two items per Big Five factor on the BFI-10, nor disposition to reward or punishment, as indexed by five items on the BIS scale and by six items on the BAS scale (with two items per subscale, i.e., fun seeking, drive, and reward responsiveness), nor current depressive or anxious mood, as indexed by two items on the PHQ-4, appear to be significant predictors of iWCST measures, including PE, in neurotypical individuals. DeYoung et al. (2005), Schretlen et al. (2010), and Stanek and Ones (2023) provide more information on possible relationships between personality and cognitive ability.

## 4 Discussion

The present study had three main goals in attempting to support further innovation in the field of neuropsychology. First, the results of the study clearly indicate that the iWCST is an appropriate tool for the unsupervised online assessment of cognitive flexibility in neurotypical individuals. Second, the results of this study, particularly the BAIN hypothesis evaluation (Hoijtink et al., 2019), provide conclusive evidence in favor of the novel GIC theory over the established SAS theory, suggesting that goal-directed instrumental control contributes significantly to cognitive flexibility. Third, the study exemplifies how the application of Bayesian data analysis can contribute to further advances in the field of neuropsychology.

### 4.1 Conclusion on the validity of the iWCST

Regarding the validity of the iWCST for assessing cognitive flexibility, it’s worth reconsidering some sample statistics. First, of the 826 individuals who responded to our email hyperlink, 503 completed the entire data collection (approximately 60.9%). Of the 323 individuals who dropped out, the majority (289 individuals, or approximately 89.5% of all dropouts) decided not to continue during the task instructions (early dropouts), while only 34 individuals (or approximately 10.5% of all dropouts) began but did not complete the data collection (late dropouts). Of these 34 late dropouts, 31 did not complete the iWCST, while the remaining three completed the iWCST but did not complete the questionnaires.

These statistics support the idea that the iWCST can collect representative samples of neurotypical individuals, since those who chose to participate after seeing the full task instructions were highly likely to complete the task. It also suggests that the cognitive load associated with the iWCST may be well tolerated by neurotypical individuals, as those who completed it appeared to do so with concentration, despite the unsupervised online data collection. Consequently, data quality was high among those who completed the iWCST and questionnaires in full, with only 14 out of 503 records (approximately 2.8%) requiring exclusion based on our pre-specified criteria for iWCST data quality.

The ESE replication, i.e., the finding that nPE% > rPE%, is another strong argument for the validity of the iWCST for assessing cognitive flexibility. This finding was previously reported for supervised paper-and-pencil (Kopp et al., 2019) and on-site computerized (Kopp et al., 2023, individual administration; Steinke et al., 2020a, group-wise administration) variants of the WCST and was replicated in this fully unsupervised online study, with slightly higher effect sizes compared to these two studies that included computerized versions of the WCST. Thus, the replicability of the ESE in neurotypical individuals has been demonstrated beyond reasonable doubt in the totality of studies that have included computerized and internet-based versions of the WCST with a total sample size of nearly 1,000 neurotypical individuals.

### 4.2 Conclusion on the theories considered

The robust behavioral ESE phenomenon consists of the simple empirical fact that non-repetitive PE (%) > repetitive PE (%) and is also important in the realm of neuropsychological theories. The reason the ESE is theoretically relevant is that the ESE supports the novel GIC theory (Kopp et al., 2023) over the established SAS theory (Norman and Shallice, 1981; Shallice, 1982; Shallice and Burgess, 1991, 1996; Shallice and Cooper, 2011). Specifically, as explained in more detail in the Introduction, SAS theory predicts nPE% < rPE%, due to the stronger involvement of the SAS in non-routine compared to routine situations, whereas GIC theory predicts nPE% > rPE%, due to the retrieval of goal-directed instrumental memory in repetitive situations only. Our confirmatory Bayesian data analysis yielded an estimated *δ* = 0.604 (point value of central tendency), with a 95 percent credible interval of [0.508–0.701], suggesting that the ordinal hypothesis nPE% > rPE% is correct. In addition, according to the BAIN hypothesis evaluation (Hoijtink et al., 2019), the posterior model probability of the SAS theory is very close to 0, while the posterior model probability of the GIC theory is very close to 1. These statistical findings are clearly inconsistent with the SAS theory, which therefore does not appear to capture well the essential cognitive processes behind the dynamic modulation of cognitive flexibility. Since a major goal of the present study was to reject the theory that received less support from the data, in accordance with the falsifiability approach, we reject the SAS theory as a viable explanation of cognitive flexibility as assessed in card-sorting tasks.

**Figure 11:**
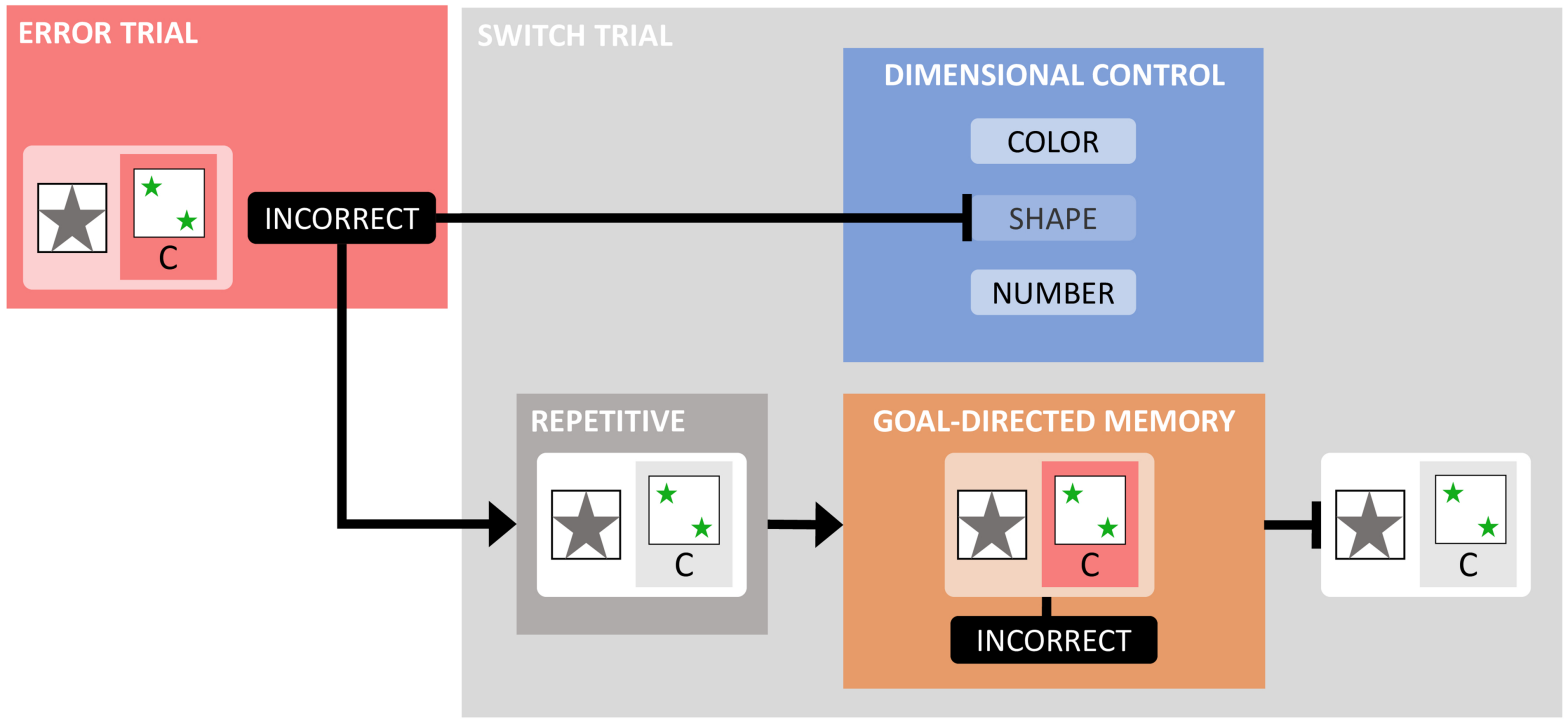
The figure illustrates cognitive processes on switch trials following informative error trials, as proposed by the theory of goal-directed instrumental control (GIC). The figure uses the SHAPE dimension, the ‘asterisk’ dimensional feature, and the C key as examples. On the error trial shown on the left, a target card containing the asterisk feature was sorted by SHAPE to the keycard containing two green asterisks by pressing the C key (see Figure 1 for the complete layout of the keycards and associated keys), and the feedback INCORRECT provided an informative error signal. The GIC theory postulates the existence of two independent cognitive processes following this error signal. Details of these processes are shown on the switch trial shown on the right. First, executive function exerts dimensional control by inhibiting the continued prioritization of the SHAPE dimension, thereby generally preventing the occurrence of PE. Second, the reactivation of goal-directed instrumental memory, contingent on the repetition of an asterisk feature on the switch trial’s target card, combined with the consideration of responding again with the C key, the reactivation of goal-directed instrumental memory facilitates the retrieval of the most recent feature-action-feedback episode (Kopp et al., 2023). The behavioral effect of retrieving this goal-directed, instrumental memory trace is to prevent the repetition of that feature-action pair (here, responding with the C key to an asterisked target card), producing the ESE with non-repetitive PE (%) > repetitive PE (%), as shown in Figure 2.

A main theoretical conclusion from the present and related data (Steinke et al., 2020a; Kopp et al., 2023) is that the GIC theory appears to provide an adequate explanation of cognitive flexibility as assessed by card-sorting tasks. Figure 11 provides a detailed representation of how the GIC theory explains the ESE. Essentially, the GIC theory posits the existence of two independent cognitive processes that follow informative error signals. First, regardless of the repetition vs. alternation of feature-action pairs on successive trials, executive control inhibits the continuation of the previously prioritized dimension following informative error signals, thereby generally preventing the occurrence of PE; indeed, we routinely find less than 10 % PE on average in neurotypical individuals in our studies. Second, goal-directed, instrumental memory traces are retrieved conditional on feature-action repetition on successive trials. In our previous study, we showed that these memory traces associate action-relevant features, actions, and feedback events from the most recent error trials (Kopp et al., 2023). The behavioral effect of retrieving traces of these instrumental episodes is to prevent the repetition of recently punished feature-action pairs, thereby producing the ESE, i.e., nPE% > rPE%. Note that the results of the exploratory Bayesian correlation/network analysis are consistent with the assumption of two independent cognitive processes following informative error signals, because categories%, SLE%, nPE%, and IE% appear to form a coherent subnetwork of iWCST measures, while rPE% may be subject to processes independent of the process governing the larger subnetwork (Figure 10).

The GIC theory has roots in dual-process theories of instrumental learning (Dickinson, 1994; De Wit and Dickinson, 2009; Balleine and O’Doherty, 2010). Dual-process theories distinguish between the formation of habitual and goal-directed memory during instrumental learning. Habits, defined as stimulus–response associations, are learned slowly and incrementally and are inflexible and insensitive to sudden changes in response-outcome contingencies (Dickinson and Pérez, 2018). Because it has proven surprisingly difficult to study habits in humans (de Wit et al., 2018), investigations of habitual control have used experimental paradigms that extensively train an instrumental response under consistent conditions. Such (over)training is thought to lead to a shift from goal-directed to habitual control. Goal-directed instrumental control is based on stimulus–response-outcome associations (Dickinson, 1994), allows for flexible adaptation to sudden changes in response-outcome contingencies, but is cognitively demanding. The field of reinforcement learning (Sutton and Barto, 2018), developed in computer science, is characterized by a similar dichotomy between model-based (resembling goal-directed) and model-free (resembling habitual) learning (Dolan and Dayan, 2013; Decker et al., 2016). The model-based system relies on internal models of the environment, allowing the simulation of possible actions and their potential outcomes based on the environmental model. This system is often associated with goal-directed decision-making, where decisions are made based on explicit consideration of expected outcomes and their associated values. The model-free system, on the other hand, is more habitual. It operates on the basis of learned associations between stimuli and responses that are driven by their outcomes, without explicit consideration of the structure of the environment. In model-free learning, decisions are made based on the reinforcement history of learned stimulus–response associations. Regarding this issue, our group has developed a computational model of the ESE, based on the distinction between model-based and model-free reinforcement learning (Steinke et al., 2018, 2020a,b,c; Steinke and Kopp, 2020).

From the paragraphs above, it should be clear that the GIC theory views the occurrence of PE as a behavioral signature of habit formation that can occasionally override executive control, even in neurotypical individuals, and the ESE as a behavioral signature of goal-directed instrumental control. This analysis ultimately leads to a three-level theory of cognitive flexibility, with the levels being habitual and goal-directed instrumental (alternatively, operational) control and executive (alternatively, strategic) control (see (Kopp, 2024), for a detailed account of a three-level theory of cognitive flexibility).

A cornerstone of this conceptualization is that retrieving goal-directed, instrumental memory traces depends on the repetition of feature-action conjunctions, as shown in Figure 11 and demonstrated in Kopp et al. (2023). Conjunctive retrieval on switch trials, which depends on the repetition of both features *and* actions, is instrumental in nature and generates the ESE. This stands in contrast to disjunctive retrieval, which on switch trials depends on the repetition of either features *or* actions and generates switch costs in cued card-sorting tasks (Kopp et al., 2020a,b).

Our integration of GIC theory within a broader framework of three levels of retrieval-based behavioral control (habitual, goal-directed/operational, executive/strategic) may contribute to a better understanding of cognitive flexibility (Kopp, 2024). GIC theory shares similarities not only with the dual-process theory of instrumental learning, but also with several retrieval-based accounts in cognitive psychology. These theories include, but are not limited to, the event coding theory (see Hommel et al., 2001), which posits that perception and action are closely linked, with representations of events encoded in a common format; the binding and retrieval theory of action control (see Frings et al., 2020), which examines how actions are bound to contexts and retrieved during action selection; learning theories of cognitive control (see Chein and Schneider, 2012), which examine how cognitive control skills are acquired through learning processes; theories linking memory and inhibition (see Anderson and Hulbert, 2021), which examine how memory processes interact with inhibitory control mechanisms; and instance theories of cognition (see Jamieson et al., 2022), which propose that cognitive processes involve the retrieval of specific instances or episodes from memory to guide current behavior. Evaluating these theories for their explanatory power may ultimately provide a comprehensive understanding of cognitive flexibility. Further neuropsychological research, particularly in patients with neurological disorders, is needed to elucidate the neuroanatomical correlates associated with repetition-dependent cognitive processes.

A related group of theories is the Bayesian view of the brain, which conceptualizes a predictive system that operates through action-perception loops using feedback-feedforward connectivity (e.g., Friston, 2010; Kolossa et al., 2015; Friston et al., 2017) and has been repeatedly applied to the Wisconsin card-sorting task (D’Alessandro et al., 2020; Barceló, 2021). From this perspective, the reduction of PE in repetitive (or stable) versus non-repetitive (or volatile) task contexts can be considered a specific example of repetition-dependent plasticity, whether simply characterized by reduced responding to repetition (e.g., Auksztulewicz and Friston, 2016) or, alternatively, by short-term memory that guides adaptation to predictable events (Jonides et al., 2008). Either way, these ideas support the notion that understanding the ESE and related phenomena requires exploring how repetition of prior stimulus–response exposures affects cognitive processes, perhaps in a manner similar to GIC theory, thereby contributing to a general understanding of cognitive flexibility.

### 4.3 Conclusion on the transition to Bayesian statistics

In our study, we distinguished between two approaches to Bayesian data analysis: confirmatory and exploratory. Confirmatory analysis focuses on hypothesis testing and theory evaluation. It uses posterior model probabilities to assess the plausibility of hypotheses and integrates prior beliefs and observed data to compare the plausibility of competing ideas. Exploratory Bayesian analysis, on the other hand, emphasizes iterative learning through parameter estimation, facilitating the cumulative discovery of new insights and relationships in the data. User-friendly tools such as JASP (JASP Team, 2024) and its BAIN module (Hoijtink et al., 2019) make Bayesian statistics more accessible. Throughout this article, we have argued that confirmatory Bayesian data analysis can further advance our theoretical understanding of cognitive processes important to neuropsychological research. We hope that this example of confirmatory Bayesian data analysis will encourage more frequent use of this highly targeted method in neuropsychological research.

## 5 Study limitations and future directions

Our study has some limitations, which are primarily related to issues of generalizability. The main limitation is that only a sample of neurotypical individuals was studied, which precludes generalization to patients with brain damage. In a previous study, the ESE was observed in consecutively sampled patients with a variety of brain diseases, but no clear disease-behavior relationship emerged (Kopp et al., 2019). Thus, understanding how the presence of neurological disease affects PE, including the ESE, is still an important area of neuropsychological research on cognitive flexibility (Dajani and Uddin, 2015). Specifically, brain-behavior relations in patients with focal brain lesions should be examined to understand which neural structures are involved in habitual, goal-directed/operational, and cognitive/strategic behavioral control for balancing cognitive flexibility against stability (Goschke and Bolte, 2014; Dreisbach and Fröber, 2019; Kopp, in press). However, the suitability of the iWCST for use in patients with neurological diseases remains to be evaluated. If the cognitive load of the iWCST is found to be appropriate for individuals with focal brain lesions, the application of voxel-based lesion-behavior mapping (Pustina and Mirman, 2022) should be the next step in elucidating the neuropsychological correlates of PE and the ESE in card-sorting tasks.

The focus of the present study is primarily on the ESE, so it would be beneficial to measure it in the presence of more prevalent PE. As mentioned above, we routinely find that neurotypical individuals have an average of less than 10 % PE, even in non-repetitive situations. A substantial proportion of the participants (*N* = 59; see Table 3) did not commit any PE during their entire iWCST assessment and, strictly speaking, it is not possible to study the ESE in the absence of PE. This issue is illustrated by the fact that after excluding these participants, the estimated effect size increases to *δ* = 0.661 (point value of central tendency), with a 95 percent credible interval of [0.557–0.765] in those participants who committed one or more PE. Follow-up studies of neurotypical individuals may well benefit from modifications of the iWCST, such as administering it in the context of dual-task management, which is known to increase the probability to commit PE, especially when the additional task administered simultaneously draws on similar cognitive resources as the card-sorting task (Cooper et al., 2012).

Most participants who provided complete records requested and received the financial compensation offered in the invitation email. This compensation appears to have been a motivating factor for participation, and it is not currently known how motivation to participate in this type of research is influenced by other motivational forces, such as contributing to the understanding of the nature of neurological diseases. The clinical application of the iWCST requires the resolution of several translational issues, such as the effects of motivational forces to participate and the appropriateness of cognitive load for individuals with brain damage. If these translational issues can be resolved, the introduction of the iWCST into clinical neuropsychology offers a number of advantages, including massively increased reach for obtaining large and more representative sample sizes and facilitated opportunities for disease-specific standardization of this important neuropsychological test (Brooks et al., 2009; Kiselica et al., 2023).

To avoid potential misunderstanding, we do not claim to have invented digital neuropsychology, theory-driven research, or Bayesian statistics within neuropsychology; indeed, there are many examples of each of these achievements in the literature. The innovation in this study lies in the unique alignment of these three building blocks, including the use of an Internet-based version of the Wisconsin card-sorting task (i.e., iWCST), the development of a diagnostic test to compare two predictions derived from different theoretical backgrounds (SAS and GIC theories), and the use of a sophisticated method of confirmatory Bayesian hypothesis testing.

## 6 Conclusion

Neuropsychology should take advantage of digital technologies. This technological advancement will create new opportunities for neuropsychological research by significantly expanding the reach of neuropsychological assessment. As demonstrated in the present study, the quality of behavioral data obtained through unsupervised online testing can reach high levels. Unprecedented opportunities for Bayesian hypothesis evaluation will be achieved with large sample sizes, accelerating the development of neuropsychological theories. Here we have shown that the established SAS theory does not adequately explain the dynamics of cognitive flexibility, whereas the novel GIC theory seems to provide a good starting point for its understanding. According to GIC theory, executive/strategic control, whose limits of efficiency can be measured as PE in non-repetitive situations, and goal-directed instrumental/operational control, whose efficiency can be measured as PE in repetitive situations and the ESE, work together to prevent the occurrence of habitual responses (Kopp, 2024). In addition, these digital technologies will provide one of the foundations for the future of clinical neuropsychology, with internet-based assessment and rehabilitation tools that will enable remote patient care, perhaps combined with supervised tele-neuropsychological techniques (Singh and Germine, 2021; Luxton et al., 2024). The iWCST presented here is just one example of how this adoption of digital technologies is likely to evolve in the future of neuropsychology.

## Data availability statement

The raw data supporting the conclusions of this article will be made available by the authors, without undue reservation.

## Ethics statement

The studies involving humans were approved by Ethikkommission Medizinische Hochschule Hannover. The studies were conducted in accordance with the local legislation and institutional requirements. The participants provided their written informed consent to participate in this study.

## Author contributions

CS: Writing – original draft, Writing – review & editing. BK: Writing – original draft, Writing – review & editing.

## Footnotes

^1^https://www.parinc.com/What-is-PARiConnect

^2^Readers may recall that the so-called Law of Effect states that behaviors followed by positive outcomes are more likely to be repeated, while behaviors followed by negative outcomes are less likely to be repeated (Thorndike, 1933). From the perspective of operant conditioning (Skinner, 1953), negative outcomes reduce the likelihood of behaviors and, by extension in GIC theory, of cognitive processes such as the continuation of a cognitive set.

^3^https://jatos.mindprobe.eu/

^4^The JASP Team, Amsterdam, NL, The Netherlands, https://jasp-stats.org/, accessed January 15, 2024.
