# Supplementary material for: The future of neuropsychology is digital, theory-driven, and Bayesian: a paradigmatic study of cognitive flexibility
Source: Front Psychol. 2024 Jul 12;15:1437192. doi: 10.3389/fpsyg.2024.1437192 (PMC11276732; doi:10.3389/fpsyg.2024.1437192)
Supplement: Supplementary file 1 [file Data_Sheet_1.DOCX]

***Online Supplementary Material***

**The future of neuropsychology is digital, theory-driven, and Bayesian:**

**A paradigmatic study of cognitive flexibility**

Clara Schmerwitz^1^, Bruno Kopp^1*^

^1^ Cognitive Neuropsychology, Department of Neurology, Hannover Medical School, Hannover, Germany

**Email**

[kopp.bruno@mh-hannover.de](mailto:kopp.bruno@mh-hannover.de)

**S1 Setting up the Online Study**


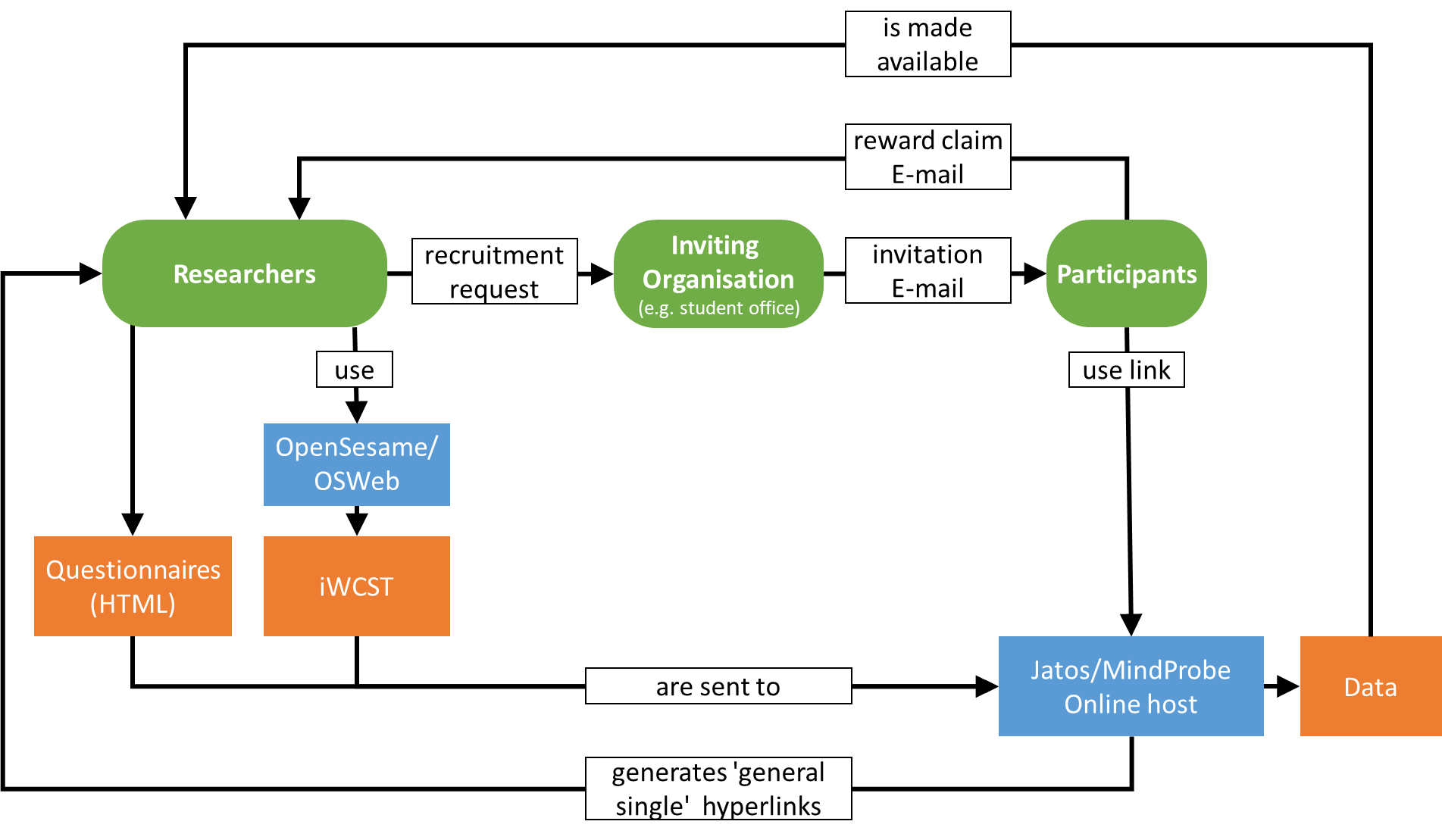


**Figure S1.1.** Workflow diagram of the online study. iWCST (internet-based Wisconsin Card-Sorting Task). Green rounded rectangles show people involved; blue rectangles represent software used; orange rectangles represent code & data files; white rectangles represent interactive processes.

**Figure S1.2.** The generic content of the invitation e-mail. The attached PDF document provided details of the privacy and anonymity practices used. Please note that after clicking on the hyperlink, respondents were first asked to confirm their informed consent to participate in the study, pending their review and acceptance of the privacy policy specified in the attached document. They were also reminded to generate a unique 8-digit alpha-numeric code for anonymous identification as part of their claim for monetary reward, which would be emailed to the researchers and could be cross-referenced with records to ensure actual study participation. GDPR, General Data Protection Regulation of the European Union (https://gdpr.eu/).

HELLO (name of university) students... Research into the connections between the brain and cognition is playing an increasingly important role in neurology.

NEUROCOGNITION ONLINE - 10€ in 30 minutes

**Hey, are you interested in neuropsychology...**

**... and want to earn some extra money?**

Then join our online neurocognition study!

Participation will take a maximum of **30 minutes** and will be **rewarded with 10€**.

If you have any questions or concerns, please email us at

[card.sorting@test.de](mailto:card.sorting@test.de).

**Important: Participation in the study is only possible with a desktop or laptop, not with touchscreen devices (such as a tablet or smartphone)!**

Please click on the following link to participate in the study:

Neurocognition Study: (hyperlink)

The research project is located at the Hannover Medical School. The anonymized data will initially be stored on a GDPR compliant server in the EU and will be deleted after 3 months. Only the MHH researchers involved will have access to the data, which will not be passed on to third parties. The data protection regulations can be found in the appendix. The aim of the study is to publish group statistical results on neurocognitive processes.

Thank you for your participation!

Best regards,

(name of researcher)


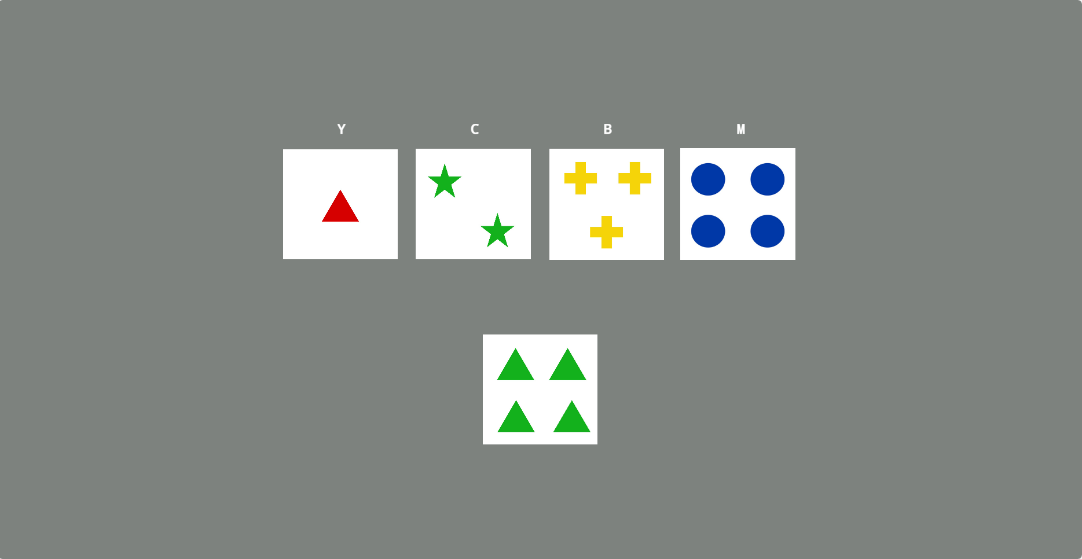


**Figure S1.3.** The stimulus arrangement of an example iWCST trial, with the four horizontally aligned keycards, including their letter keys, and a target card to be sorted appearing in a central location below the keycards. In this trial, the Y key (shared 'triangle' feature of the SHAPE dimension), the C key (shared 'green' feature of the COLOR dimension), and the M key (shared 'four' feature of the NUMBER dimension) are viable responses, while responding with the B key represents an odd error (this keycard and the depicted target card do not share any feature).

**S2 Description of the iWCST Instructions**


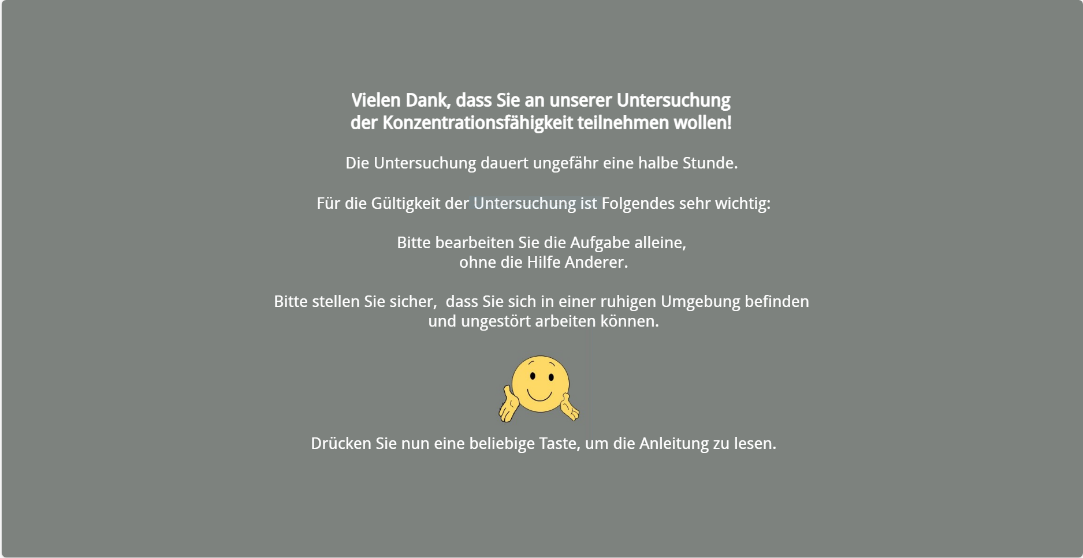


**Figure S2.1.** First task instruction slide. Translated from German language:

“Thank you for taking part in our study of the ability to concentrate! The assessment takes about half an hour. The following is very important for the validity of the assessment: Please complete the task alone, without the help of others. Please ensure that you are in a quiet environment and can work undisturbed.

Press any key to read the instructions.”


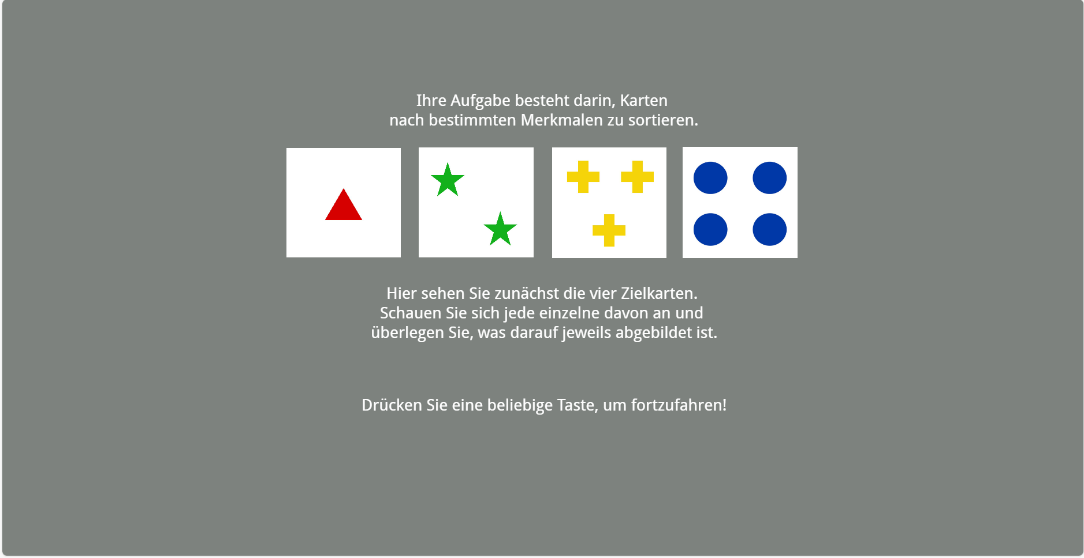


**Figure S2.2.** Second task instruction slide. Translated from German language:

“Your task is to sort cards according to certain characteristics. […]

Here are the four keycards. Look at each one individually and think about what is depicted on each one.

Press any key to continue!”


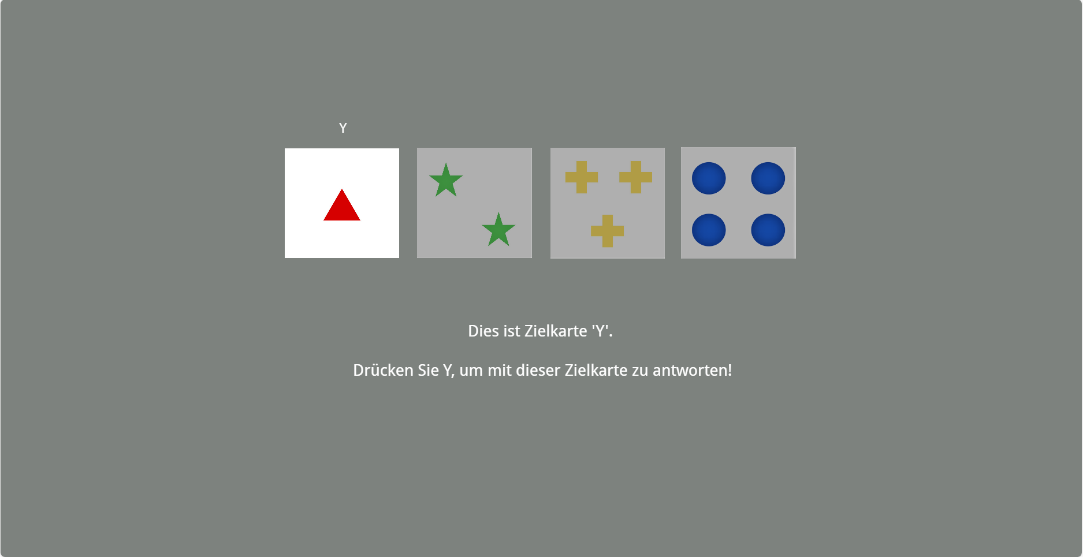


**Figure S2.3.** Third task instruction slide. Translated from German language:

“This is keycard 'Y'. Press the Y key to respond with this keycard!”


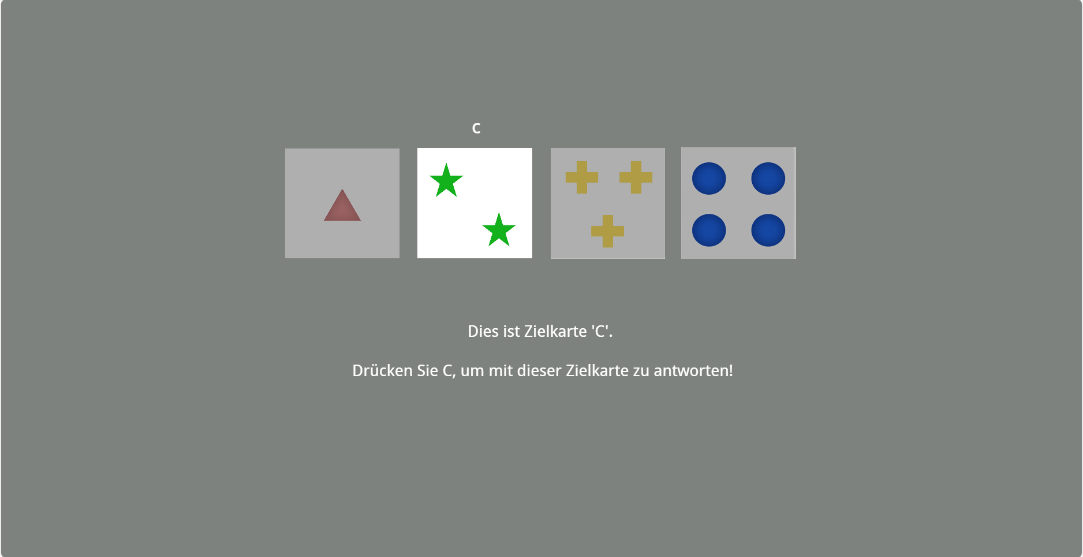


**Figure S2.4.** Fourth task instruction slide. Translated from German language:

“This is keycard 'C'. Press the C key to respond with this keycard!”


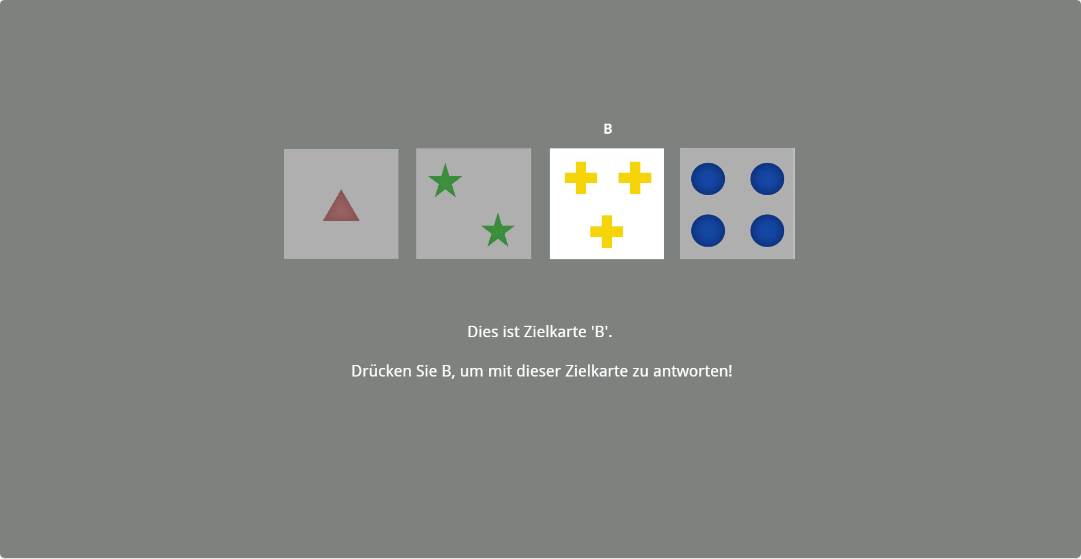


**Figure S2.5.** Fifth task instruction slide. Translated from German language:

“This is keycard 'B'. Press the B key to respond with this keycard!”


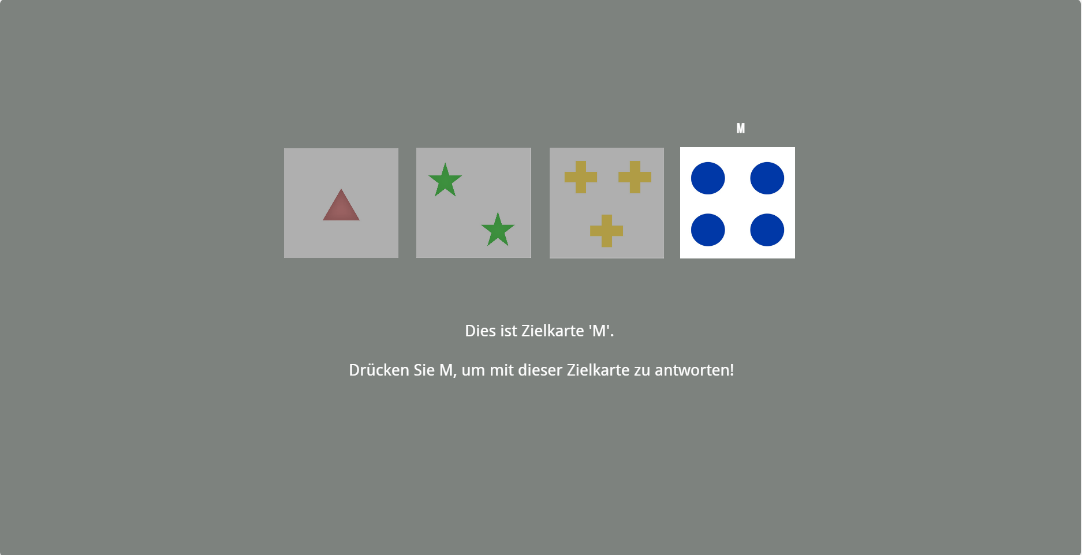


**Figure S2.6.** Sixth task instruction slide. Translated from German language:

“This is keycard 'M'. Press the M key to respond with this keycard!”


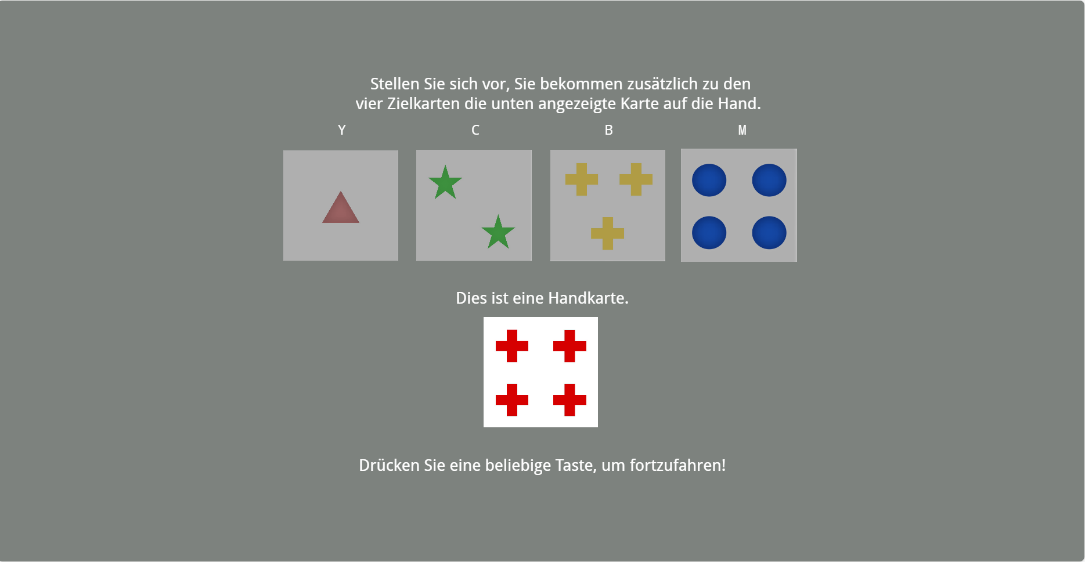


**Figure S2.7.** Seventh task instruction slide. Translated from German language:

“Imagine that in addition to the four keycards, you are also handling a target card as shown below the four keycards. […]

This is a target card. […]

Press any key to continue!”


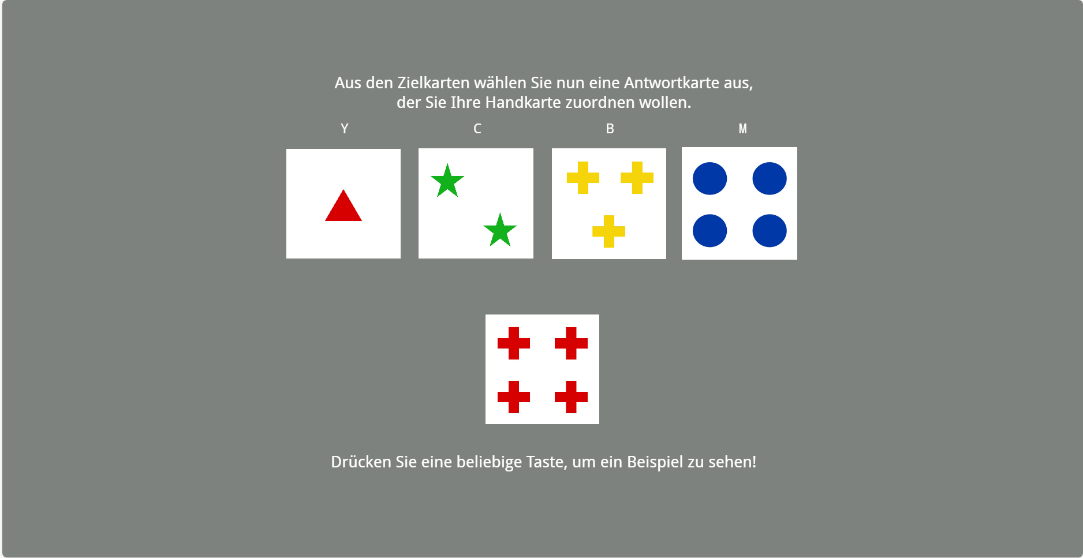


**Figure S2*.*8.** Eighth task instruction slide. Translated from German language:

“Now select the keycard to which you would like to assign your target card. […]

Press any key to see an example!”


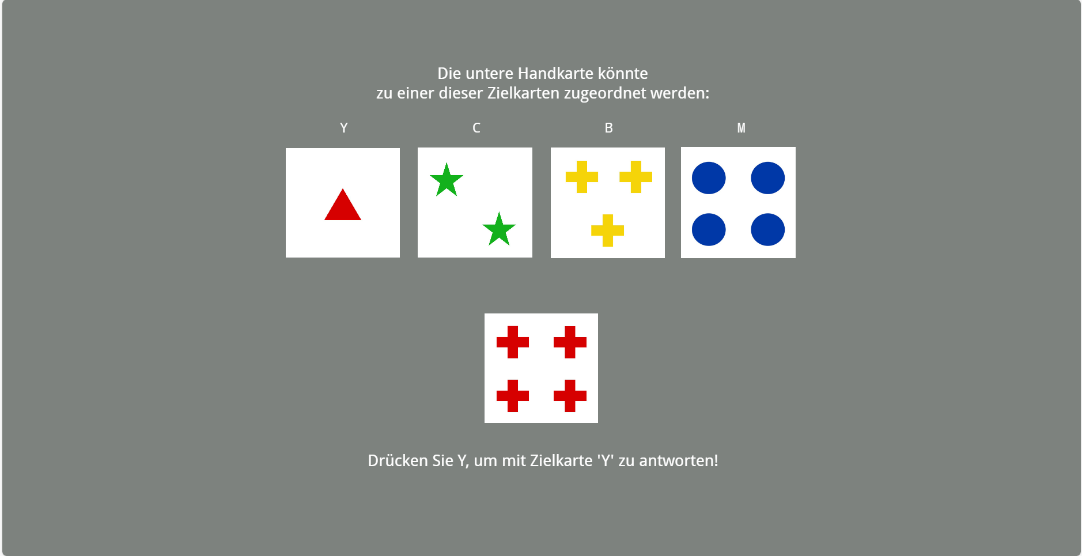


**Figure S2*.*9.** Ninth task instruction slide. Translated from German language:

“The bottom target card could be assigned to one of these keycards: […]

Press the Y key to respond with keycard 'Y'!”


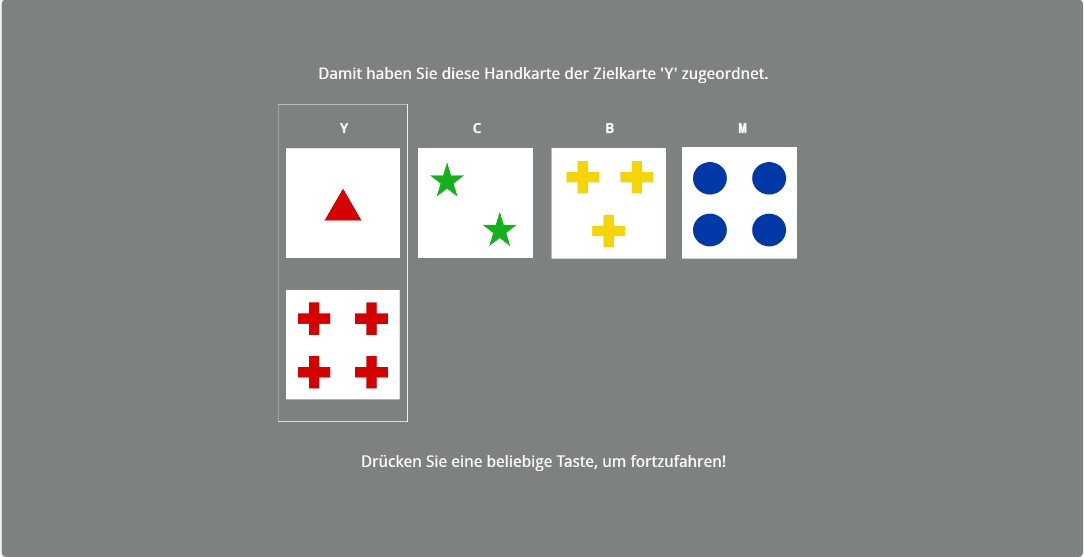


**Figure S2*.*10.** Tenth task instruction slide. Translated from German language:

“You have now assigned this target card to the keycard 'Y'. […]

Press any key to continue!”


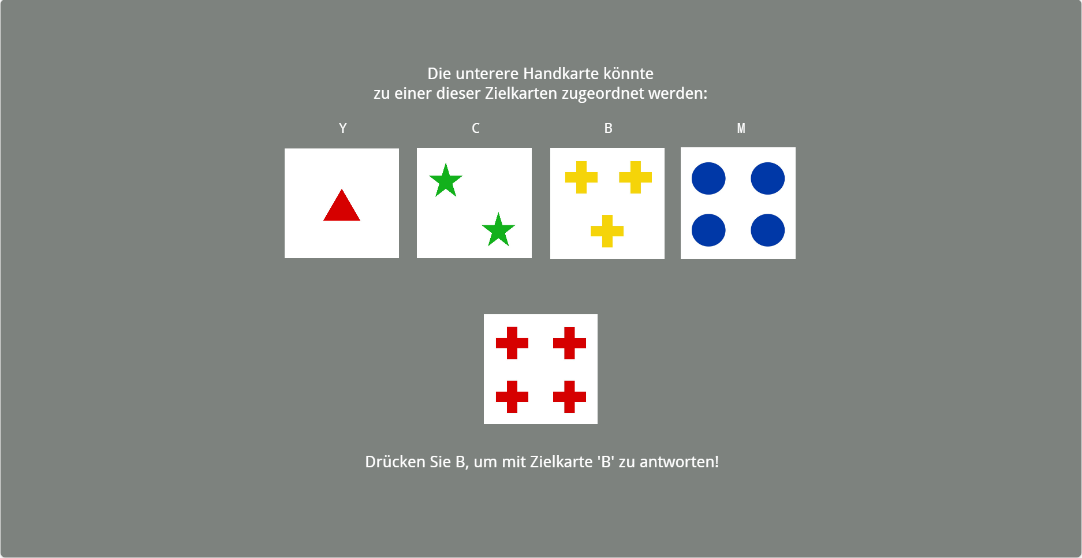


**Figure S2.11.** Eleventh task instruction slide. Translated from German language:

“The bottom target card could be assigned to one of these keycards: […]

Press the B key to respond with keycard 'B'!”


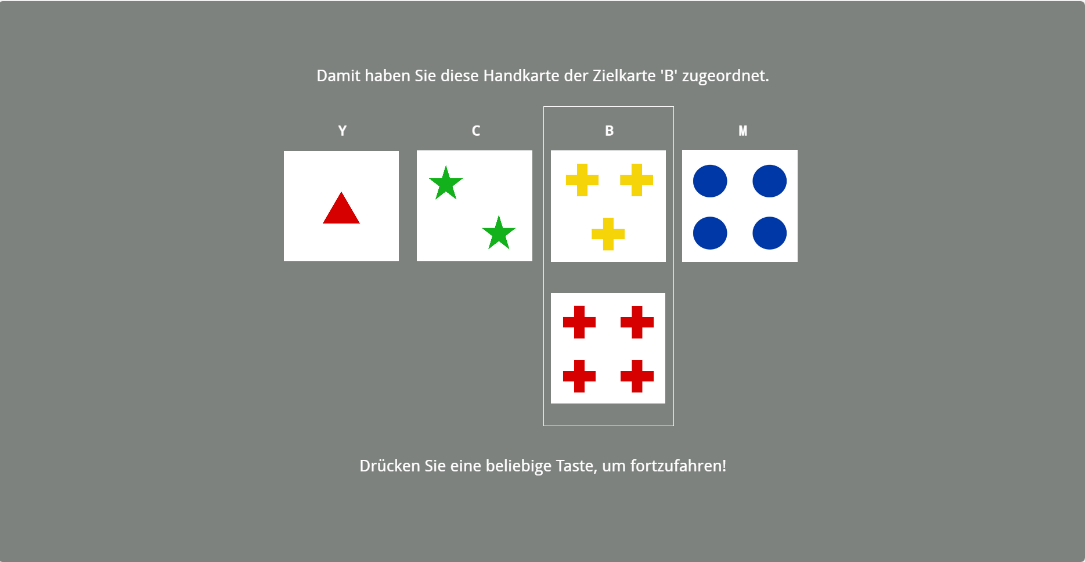


**Figure S2*.*12.** Twelveth task instruction slide. Translated from German language:

“You have now assigned this target card to the keycard 'B'. […]

Press any key to continue!”


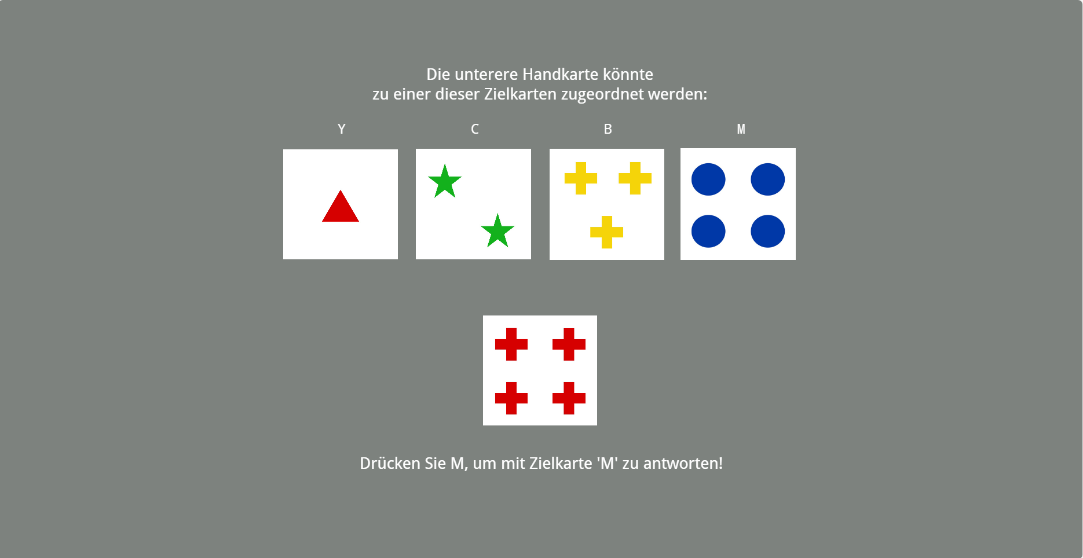


**Figure S2*.*13.** 13th task instruction slide. Translated from German language:

“The bottom target card could be assigned to one of these keycards: […]

Press the M key to respond with keycard 'M'!”


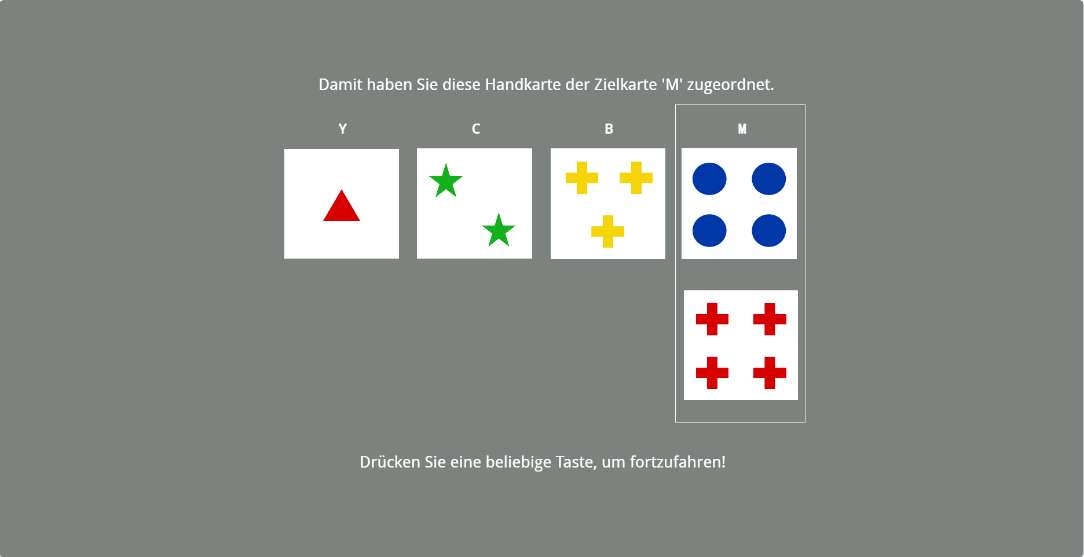


**Figure S2*.*14.** 14th task instruction slide. Translated from German language:

“You have now assigned this target card to the keycard 'M'. […]

Press any key to continue!”


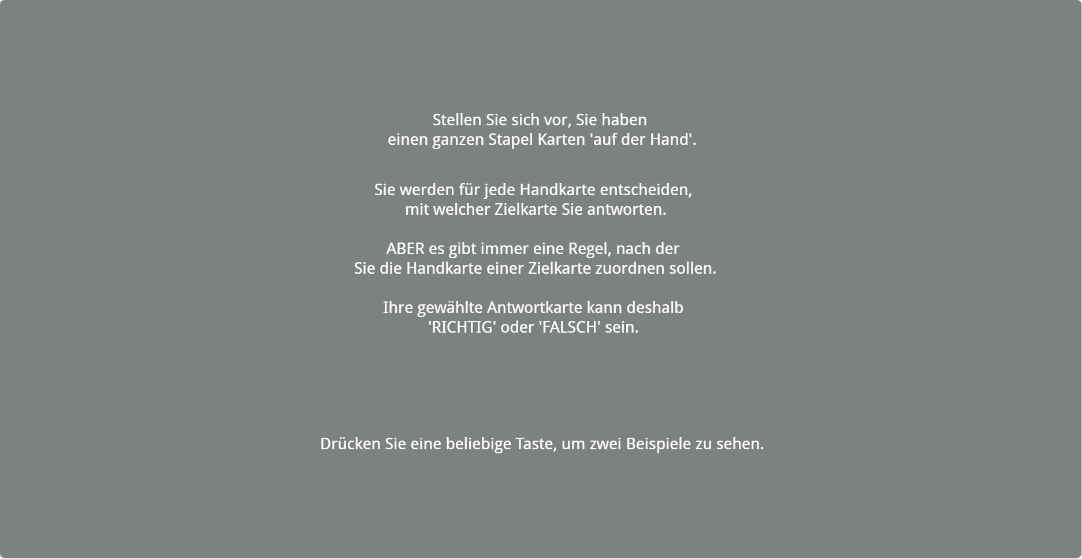


**Figure S2*.*15.** 15th task instruction slide. Translated from German language:

“Imagine that you have a whole stack of target cards 'in your hand'. For each target card, you decide which keycard to respond with.

BUT there is always a dimension according to which you should match the target card to a keycard. So the keycard you choose can be either 'CORRECT' or 'WRONG'.

Press any key to see two examples.”


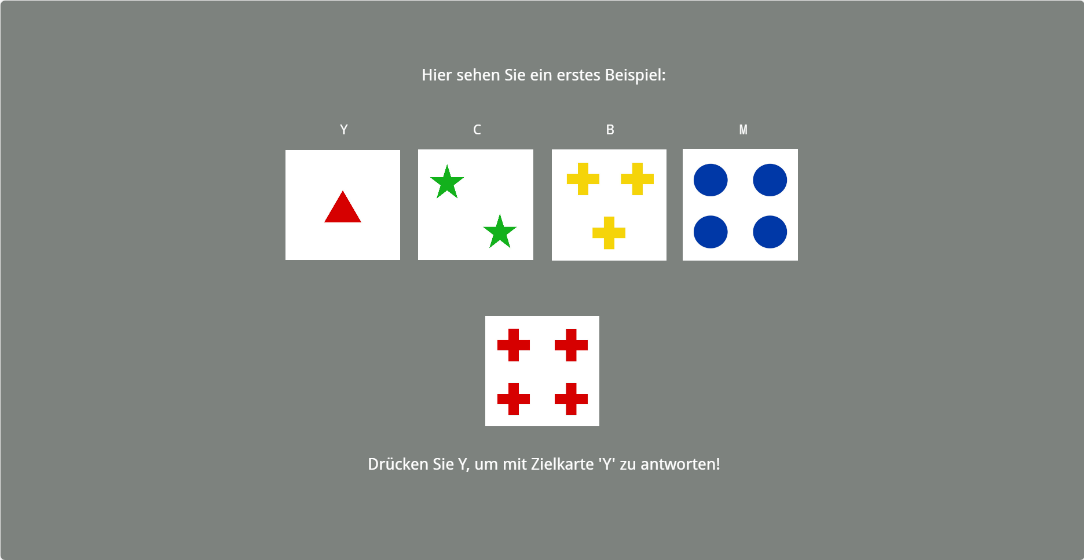


**Figure S2.16.** 16th task instruction slide. Translated from German language:

“Here you can see a first example: […]

Press the Y key to respond with target card 'Y'!”


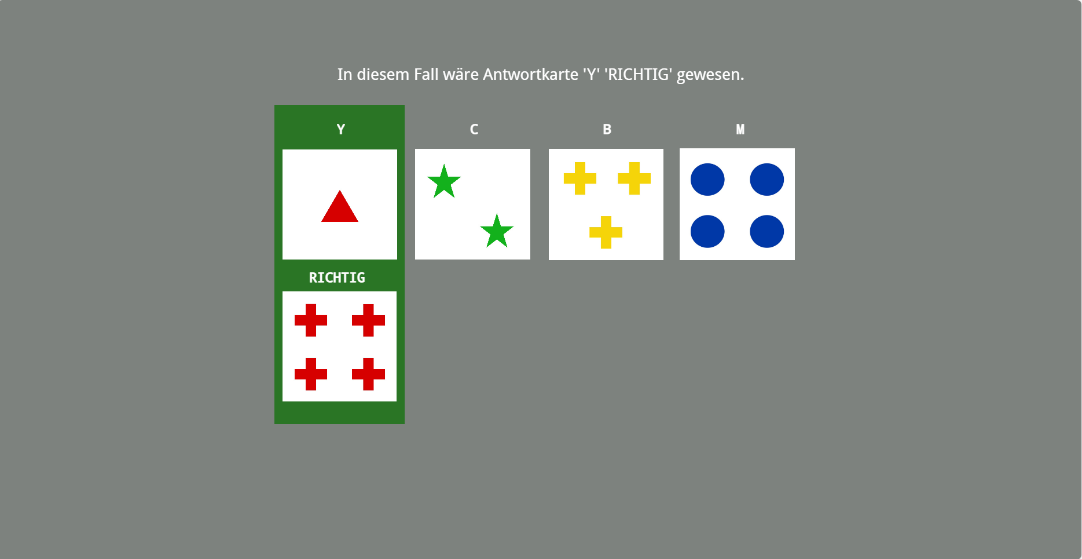


**Figure S2.17.** 17th task instruction slide. Translated from German language:

“In this case, keycard 'Y' would have been 'CORRECT'.”


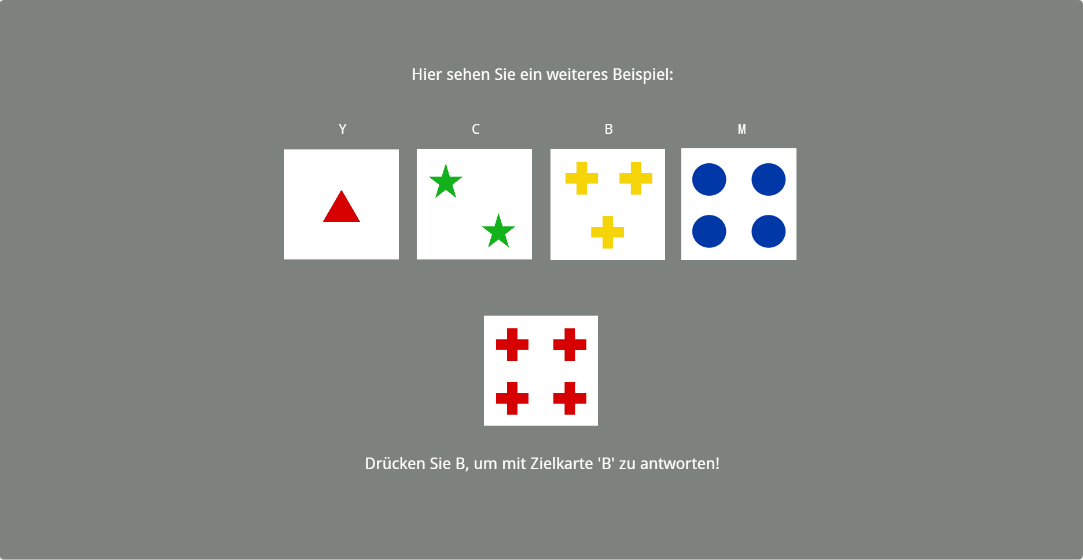


**Figure S2.18.** 18th task instruction slide. Translated from German language:

“Here you can see another example: […]

Press the B key to respond with keycard 'B'!”


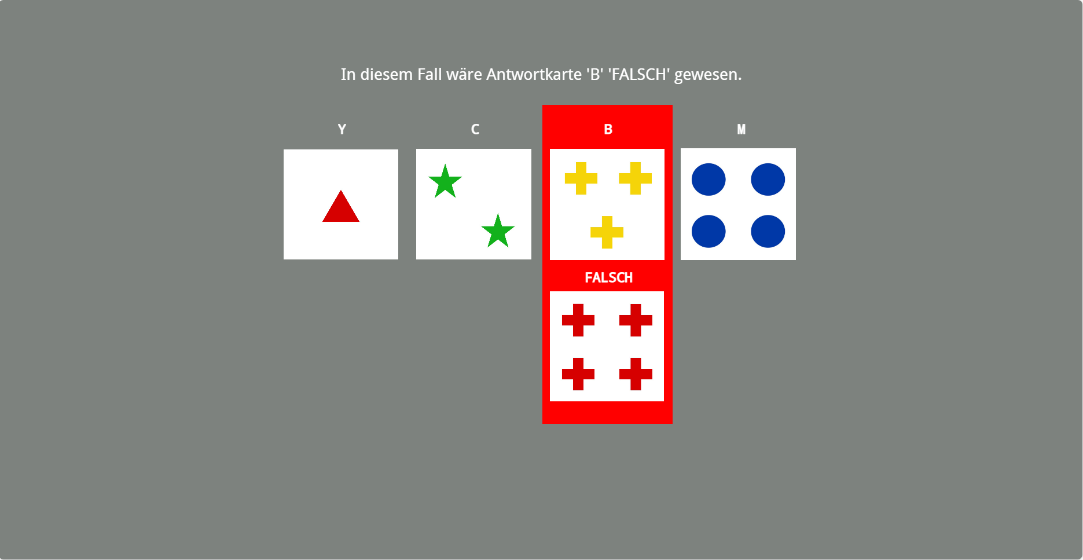


**Figure S2*.*19.** 19th task instruction slide. Translated from German language:

“In this case, keycard 'B' would have been 'WRONG'.”


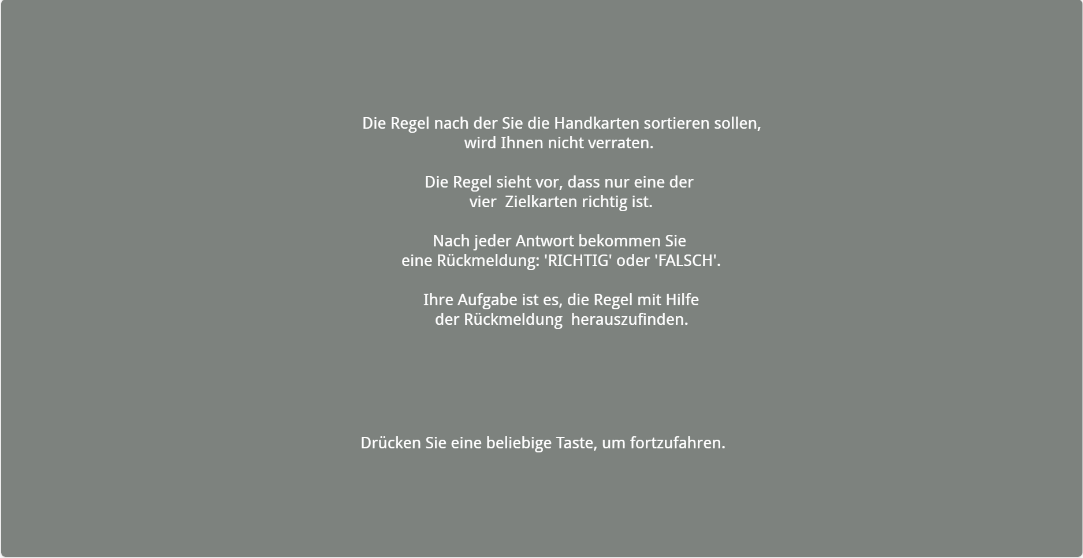


**Figure S2.20.** 20th task instruction slide. Translated from German language:

“The dimension by which you should sort the target cards will not be revealed to you. However, the dimension indicates that only one of the four keycards is correct.

You will receive feedback after each single response: 'CORRECT' or 'WRONG'.

Your task is to use these feedbacks to determine the correct dimension.

Press any key to continue!”


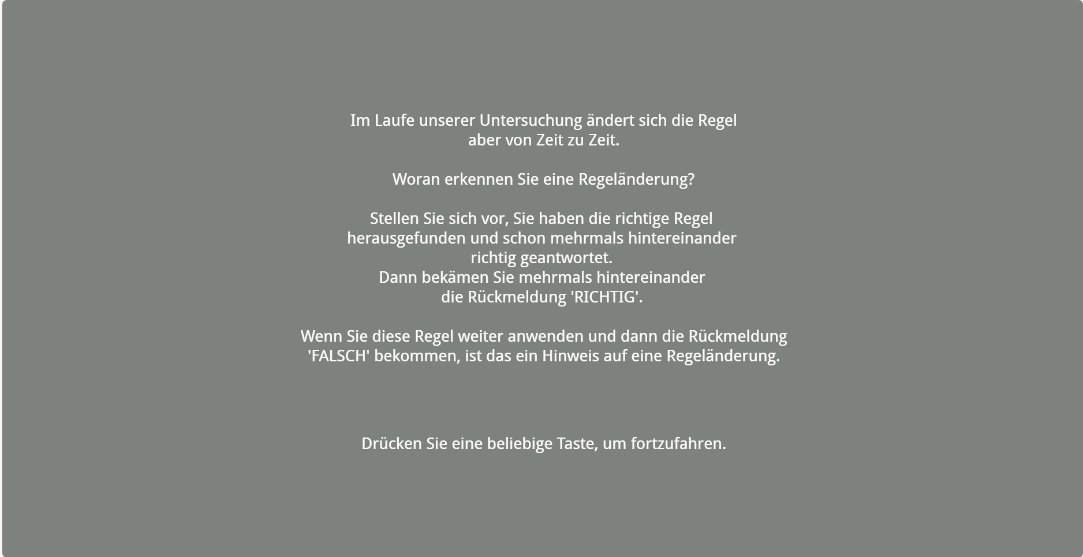


**Figure S2*.*21.** 21th task instruction slide. Translated from German language:

“In the course of our investigation, the correct dimension changes from time to time. How do you detect a change in dimension?

Imagine that you have discovered the correct dimension and have responded correctly several times in a row. Then you would receive the feedback 'CORRECT' several times in a row.

If you continue to use that dimension and then receive the feedback 'WRONG', this is an indication of a dimensional shift.

Press any key to continue!”


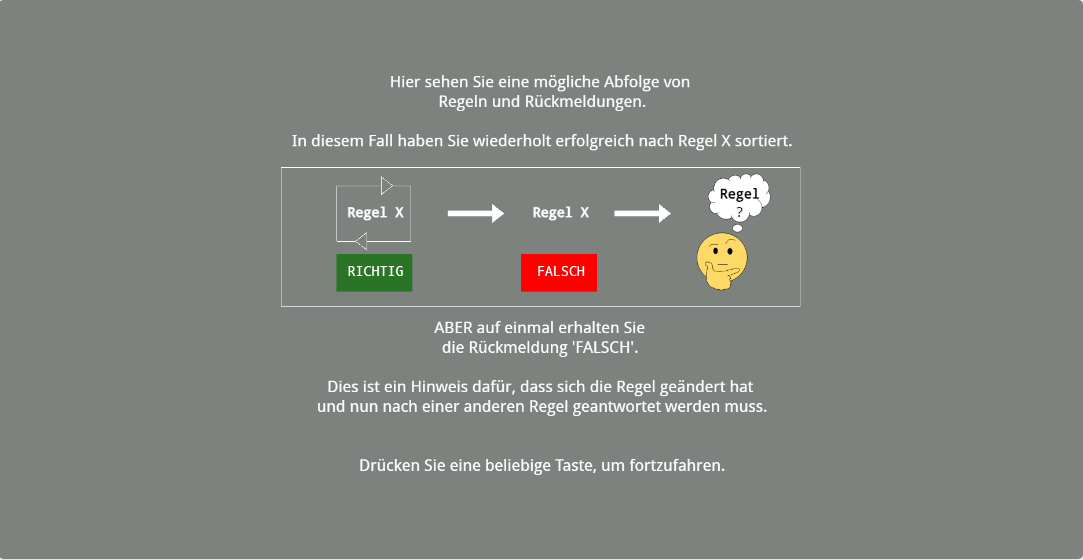


**Figure S2.22.** 22nd task instruction slide. Translated from German language:

“Here is a possible sequence of dimensions and feedback.

In this case, you have repeatedly sorted successfully by dimension X. [...]

BUT suddenly you receive the feedback 'WRONG'.

This is an indication that the correct dimension has changed and you must now respond according to another dimension.

Press any key to continue.”


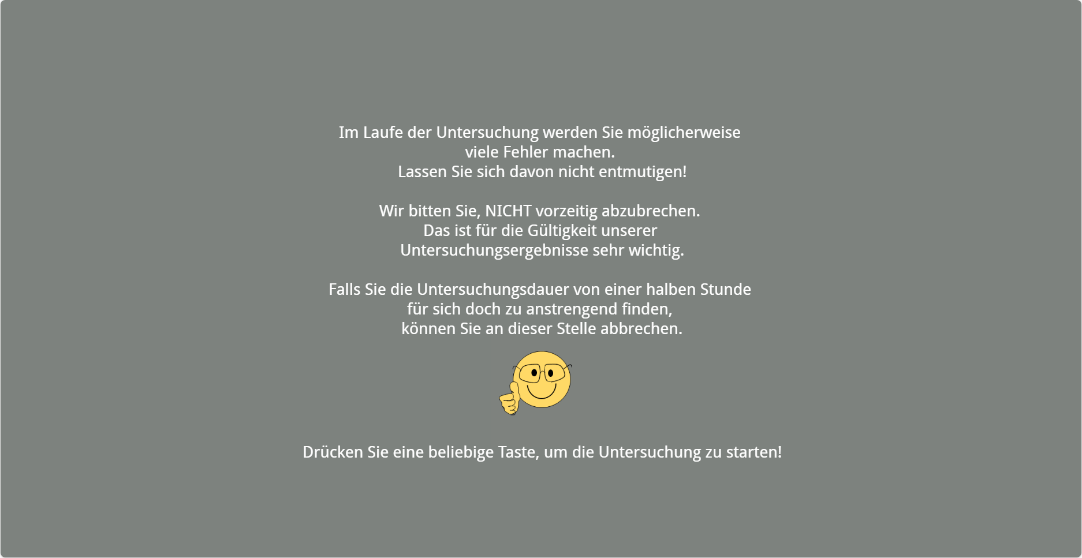


**Figure S2.23.** 23rd task instruction slide. Translated from German language:

“You may make many mistakes during the assessment. Do not be discouraged!

We ask you NOT to give up prematurely. This is very important for the validity of our study results.

If you find the half hour assessment too strenuous, you may stop at this point.

Press any key to start the assessment!”

**S3 Additional Statistics**

**S3.1. Bayesian Network Analysis of iWCST Variables**

**Table S3.1.1.** Standardized centrality measures per variable

|  | **Centrality measure** | | |
| --- | --- | --- | --- |
| **Variable** | **Betweenness** | **Closeness** | **Strength** |
| categories% | 1.79 | 1.30 | 1.36 |
| SLE% | -0.45 | 0.58 | 0.70 |
| nPE% | -0.45 | -0.11 | -0.35 |
| rPE% | -0.45 | -1.33 | -1.08 |
| IE% | -0.45 | -0.43 | -0.63 |

*Notes*. categories%, percentage of categories; SLE%, percentage of set-loss errors; nPE%, percentage of non-repetitive perseveration errors; rPE%, percentage of repetitive perseveration errors; IE%, percentage of integration errors.

**S3.2. Bayesian Regression (iWCST Variables and Questionnaires)**

**Table S3.2.1.** Bayesian model comparison for the iWCST variable categories%

| **Models** | ***p*(M)** | ***p*(M\|data)** | ***BF_M_*** | ***BF_10_*** | ***R²*** | ***VIF*** |
| --- | --- | --- | --- | --- | --- | --- |
| BFI-10 |  |  |  |  |  |  |
| Null model | 0.03 | 0.22 | 8.57 | 1.00 | 0.00 |  |
| Openness | 0.03 | 0.15 | 5.39 | 0.68 | 0.01 | 1.02 |
| Conscientiousness | 0.03 | 0.11 | 3.78 | 0.50 | 0.01 | 1.03 |
| Neuroticism | 0.03 | 0.03 | 1.04 | 0.15 | 0.00 | 1.03 |
| Agreeableness | 0.03 | 0.02 | 0.77 | 0.11 | 0.00 | 1.01 |
| Extroversion | 0.03 | 0.02 | 0.72 | 0.11 | 0.00 | 1.02 |
| BIS/BAS-11 |  |  |  |  |  |  |
| Null model | 0.06 | 0.50 | 14.68 | 1.00 | 0.00 |  |
| BAS-FS | 0.06 | 0.16 | 2.89 | 0.33 | 0.01 | 1.01 |
| BIS Total | 0.06 | 0.07 | 1.12 | 0.14 | 0.00 | 1.01 |
| BAS-RR | 0.06 | 0.07 | 1.10 | 0.14 | 0.00 | 1.19 |
| BAS-D | 0.06 | 0.05 | 0.80 | 0.10 | 0.00 | 1.20 |
| PHQ-4 |  |  |  |  |  |  |
| Null model | 0.25 | 0.68 | 6.38 | 1.00 | 0.00 |  |
| PHQ Anxiety | 0.25 | 0.20 | 0.74 | 0.29 | 0.00 | 1.71 |
| PHQ Depression | 0.25 | 0.09 | 0.30 | 0.13 | 0.00 | 1.71 |

*Notes.* The table presents results only for the null model and single predictor models for simplicity. These models were chosen to assess the contribution of individual predictors. VIF, variance inflation factor, calculated in classical linear regression as suggested by van den Bergh et al. (2021).

**Table S3.2.2.** Posterior summaries of regression coefficients with regard to the iWCST variable categories% and BFI-10 scores

|  | | | | | | | | **95% Credible Interval** | |
| --- | --- | --- | --- | --- | --- | --- | --- | --- | --- |
| **Coefficient** | ***p*(incl)** | ***p(*excl)** | ***p*(incl\|data)** | ***p*(excl\|data)** | ***BF_inclusion_*** | ***M*** | ***SD*** | ***lower*** | ***upper*** |
| Intercept | 1.00 | 0.00 | 1.00 | 0.00 | 1.00 | 11.52 | 0.06 | 11.41 | 11.63 |
| Extroversion | 0.50 | 0.50 | 0.15 | 0.85 | 0.17 | 0.00 | 0.03 | -0.07 | 0.03 |
| Agreeableness | 0.50 | 0.50 | 0.15 | 0.85 | 0.18 | 0.00 | 0.02 | -0.04 | 0.02 |
| Conscientiousness | 0.50 | 0.50 | 0.44 | 0.56 | 0.79 | 0.04 | 0.05 | 0.00 | 0.15 |
| Neuroticism | 0.50 | 0.50 | 0.17 | 0.83 | 0.20 | 0.01 | 0.02 | -0.01 | 0.07 |
| Openness | 0.50 | 0.50 | 0.51 | 0.49 | 1.03 | -0.04 | 0.05 | -0.14 | 0.00 |

**Table S3.2.3.** Posterior summaries of regression coefficients with regard to the iWCST variable categories% and BIS/BAS-11 scores

|  | | | | | | | | **95% Credible Interval** | |
| --- | --- | --- | --- | --- | --- | --- | --- | --- | --- |
| **Coefficient** | ***p*(incl)** | ***p(*excl)** | ***p*(incl\|data)** | ***p*(excl\|data)** | ***BF_inclusion_*** | ***M*** | ***SD*** | ***lower*** | ***upper*** |
| Intercept | 1.00 | 0.00 | 1.00 | 0.00 | 1.00 | 11.52 | 0.06 | 11.42 | 11.64 |
| BIS Total | 0.50 | 0.50 | 0.15 | 0.85 | 0.17 | 0.00 | 0.01 | 0.00 | 0.04 |
| BAS-FS | 0.50 | 0.50 | 0.28 | 0.72 | 0.39 | -0.02 | 0.04 | -0.12 | 0.00 |
| BAS-D | 0.50 | 0.50 | 0.11 | 0.89 | 0.13 | 0.00 | 0.02 | -0.01 | 0.06 |
| BAS-RR | 0.50 | 0.50 | 0.15 | 0.85 | 0.17 | 0.01 | 0.03 | 0.00 | 0.08 |

*Notes.* -FS, Fun Seeking; -D, Drive; -RR, Reward Responsiveness.

**Table S3.2.4.** Posterior summaries of regression coefficients with regard to the iWCST variable categories% and PHQ-4 scores

|  | | | | | | | | **95% Credible Interval** | |
| --- | --- | --- | --- | --- | --- | --- | --- | --- | --- |
| **Coefficient** | ***p*(incl)** | ***p(*excl)** | ***p*(incl\|data)** | ***p*(excl\|data)** | ***BF_inclusion_*** | ***M*** | ***SD*** | ***lower*** | ***upper*** |
| Intercept | 1.00 | 0.00 | 1.00 | 0.00 | 1.00 | 11.524 | 0.06 | 11.41 | 11.62 |
| PHQ Depression | 0.50 | 0.50 | 0.12 | 0.88 | 0.14 | 0.00 | 0.02 | -0.07 | 0.00 |
| PHQ Anxiety | 0.50 | 0.50 | 0.23 | 0.77 | 0.30 | -0.01 | 0.03 | -0.08 | 0.00 |

**Table S3.2.5.** Bayesian model comparison for the iWCST variable SLE%

| **Models** | ***p*(M)** | ***p*(M\|data)** | ***BF_M_*** | ***BF_10_*** | ***R²*** | ***VIF*** |
| --- | --- | --- | --- | --- | --- | --- |
| BFI-10 |  |  |  |  |  |  |
| Null model | 0.03 | 0.20 | 7.50 | 1.00 | 0.00 |  |
| Conscientiousness | 0.03 | 0.28 | 11.82 | 1.42 | 0.01 | 1.03 |
| Openness | 0.03 | 0.04 | 1.41 | 0.22 | 0.00 | 1.02 |
| Extroversion | 0.03 | 0.02 | 0.71 | 0.12 | 0.00 | 1.02 |
| Agreeableness | 0.03 | 0.02 | 0.66 | 0.12 | 0.00 | 1.01 |
| Neuroticism | 0.03 | 0.02 | 0.64 | 0.10 | 0.00 | 1.03 |
| BIS/BAS-11 |  |  |  |  |  |  |
| Null model | 0.06 | 0.19 | 3.42 | 1.00 | 0.00 |  |
| BAS-FS | 0.06 | 0.41 | 10.33 | 2.20 | 0.01 | 1.01 |
| BAS-RR | 0.06 | 0.03 | 0.50 | 0.17 | 0.00 | 1.19 |
| BAS-D | 0.06 | 0.03 | 0.42 | 0.15 | 0.00 | 1.20 |
| BIS Total | 0.06 | 0.02 | 0.29 | 0.10 | 0.00 | 1.01 |
| PHQ-4 |  |  |  |  |  |  |
| Null model | 0.25 | 0.68 | 6.29 | 1.00 | 0.00 |  |
| PHQ Anxiety | 0.25 | 0.18 | 0.67 | 0.27 | 0.00 | 1.71 |
| PHQ Depression | 0.25 | 0.11 | 0.38 | 0.17 | 0.00 | 1.71 |

*Notes.* The table presents results only for the null model and single predictor models for simplicity. These models were chosen to assess the contribution of individual predictors. VIF, variance inflation factor, calculated in classical linear regression as suggested by van den Bergh et al. (2021).

**Table S3.2.6.** Posterior summaries of regression coefficients with regard to the iWCST variable SLE% and BFI-10 scores

|  | | | | | | | | **95% Credible Interval** | |
| --- | --- | --- | --- | --- | --- | --- | --- | --- | --- |
| **Coefficient** | ***p*(incl)** | ***p(*excl)** | ***p*(incl\|data)** | ***p*(excl\|data)** | ***BF_inclusion_*** | ***M*** | ***SD*** | ***lower*** | ***upper*** |
| Intercept | 1.00 | 0.00 | 1.00 | 0.00 | 1.00 | 5.08 | 0.15 | 4.80 | 5.35 |
| Extroversion | 0.50 | 0.50 | 0.17 | 0.83 | 0.20 | 0.02 | 0.08 | 0.00 | 0.28 |
| Agreeableness | 0.50 | 0.50 | 0.15 | 0.85 | 0.17 | 0.01 | 0.04 | -0.02 | 0.15 |
| Conscientiousness | 0.50 | 0.50 | 0.66 | 0.34 | 1.94 | -0.19 | 0.17 | -0.47 | 0.00 |
| Neuroticism | 0.50 | 0.50 | 0.14 | 0.86 | 0.16 | 0.00 | 0.04 | -0.07 | 0.15 |
| Openness | 0.50 | 0.50 | 0.28 | 0.72 | 0.39 | 0.04 | 0.09 | 0.00 | 0.29 |

**Table S3.2.7.** Posterior summaries of regression coefficients with regard to the iWCST variable SLE% and BIS/BAS-11 scores

|  | | | | | | | | **95% Credible Interval** | |
| --- | --- | --- | --- | --- | --- | --- | --- | --- | --- |
| **Coefficient** | ***p*(incl)** | ***p(*excl)** | ***p*(incl\|data)** | ***p*(excl\|data)** | ***BF_inclusion_*** | ***M*** | ***SD*** | ***lower*** | ***upper*** |
| Intercept | 1.00 | 0.00 | 1.00 | 0.00 | 1.00 | 5.08 | 0.15 | 4.80 | 5.37 |
| BIS Total | 0.50 | 0.50 | 0.13 | 0.87 | 0.15 | 0.00 | 0.02 | -0.03 | 0.07 |
| BAS-FS | 0.50 | 0.50 | 0.72 | 0.28 | 2.58 | 0.21 | 0.17 | 0.00 | 0.48 |
| BAS-D | 0.50 | 0.50 | 0.15 | 0.85 | 0.18 | -0.01 | 0.06 | -0.20 | 0.07 |
| BAS-RR | 0.50 | 0.50 | 0.21 | 0.79 | 0.26 | -0.03 | 0.09 | -0.33 | 0.01 |

*Notes.* -FS, Fun Seeking; -D, Drive; -RR, Reward Responsiveness.

**Table S3.2.8.** Posterior summaries of regression coefficients with regard to the iWCST variable SLE% and PHQ-4 scores

|  | | | | | | | | **95% Credible Interval** | |
| --- | --- | --- | --- | --- | --- | --- | --- | --- | --- |
| **Coefficient** | ***p*(incl)** | ***p(*excl)** | ***p*(incl\|data)** | ***p*(excl\|data)** | ***BF_inclusion_*** | ***M*** | ***SD*** | ***lower*** | ***upper*** |
| Intercept | 1.00 | 0.00 | 1.00 | 0.00 | 1.00 | 5.08 | 0.15 | 4.77 | 5.38 |
| PHQ Depression | 0.50 | 0.50 | 0.14 | 0.86 | 0.16 | 0.01 | 0.05 | 0.00 | 0.20 |
| PHQ Anxiety | 0.50 | 0.50 | 0.21 | 0.79 | 0.27 | 0.03 | 0.07 | -0.01 | 0.21 |

**Table S3.2.9.** Bayesian model comparison for the iWCST variable nPE%

| **Models** | ***p*(M)** | ***p*(M\|data)** | ***BF_M_*** | ***BF_10_*** | ***R²*** | ***VIF*** |
| --- | --- | --- | --- | --- | --- | --- |
| BFI-10 |  |  |  |  |  |  |
| Null model | 0.03 | 0.42 | 22.62 | 1.00 | 0.00 |  |
| Openness | 0.03 | 0.11 | 3.75 | 0.26 | 0.00 | 1.02 |
| Neuroticism | 0.03 | 0.08 | 2.86 | 0.20 | 0.00 | 1.03 |
| Extroversion | 0.03 | 0.07 | 2.28 | 0.16 | 0.00 | 1.02 |
| Conscientiousness | 0.03 | 0.05 | 1.57 | 0.11 | 0.00 | 1.03 |
| Agreeableness | 0.03 | 0.04 | 1.41 | 0.10 | 0.00 | 1.01 |
| BIS/BAS-11 |  |  |  |  |  |  |
| Null model | 0.06 | 0.25 | 4.91 | 1.00 | 0.00 |  |
| BAS-D | 0.06 | 0.32 | 6.91 | 1.28 | 0.01 | 1.20 |
| BAS-FS | 0.06 | 0.04 | 0.66 | 0.17 | 0.00 | 1.01 |
| BAS-RR | 0.06 | 0.03 | 0.39 | 0.10 | 0.00 | 1.19 |
| BIS Total | 0.06 | 0.03 | 0.38 | 0.10 | 0.00 | 1.01 |
| PHQ-4 |  |  |  |  |  |  |
| Null model | 0.25 | 0.73 | 8.29 | 1.00 | 0.00 |  |
| PHQ Anxiety | 0.25 | 0.16 | 0.57 | 0.22 | 0.00 | 1.71 |
| PHQ Depression | 0.25 | 0.08 | 0.25 | 0.11 | 0.00 | 1.71 |

*Notes.* The table presents results only for the null model and single predictor models for simplicity. These models were chosen to assess the contribution of individual predictors. VIF, variance inflation factor, calculated in classical linear regression as suggested by van den Bergh et al. (2021).

**Table S3.2.10.** Posterior summaries of regression coefficients with regard to the iWCST variable nPE% and BFI-10 scores

|  | | | | | | | | **95% Credible Interval** | |
| --- | --- | --- | --- | --- | --- | --- | --- | --- | --- |
| **Coefficient** | ***p*(incl)** | ***p(*excl)** | ***p*(incl\|data)** | ***p*(excl\|data)** | ***BF_inclusion_*** | ***M*** | ***SD*** | ***lower*** | ***upper*** |
| Intercept | 1.00 | 0.00 | 1.00 | 0.00 | 1.00 | 9.24 | 0.37 | 8.52 | 9.93 |
| Extroversion | 0.50 | 0.50 | 0.17 | 0.83 | 0.21 | -0.06 | 0.22 | -0.75 | 0.10 |
| Agreeableness | 0.50 | 0.50 | 0.12 | 0.88 | 0.14 | -0.01 | 0.10 | -0.33 | 0.06 |
| Conscientiousness | 0.50 | 0.50 | 0.14 | 0.86 | 0.16 | 0.02 | 0.12 | -0.15 | 0.41 |
| Neuroticism | 0.50 | 0.50 | 0.20 | 0.80 | 0.25 | -0.07 | 0.18 | -0.67 | 0.01 |
| Openness | 0.50 | 0.50 | 0.24 | 0.76 | 0.31 | 0.09 | 0.21 | -0.07 | 0.59 |

**Table S3.2.11.** Posterior summaries of regression coefficients with regard to the iWCST variable nPE% and BIS/BAS-11 scores

|  | | | | | | | | **95% Credible Interval** | |
| --- | --- | --- | --- | --- | --- | --- | --- | --- | --- |
| **Coefficient** | ***p*(incl)** | ***p(*excl)** | ***p*(incl\|data)** | ***p*(excl\|data)** | ***BF_inclusion_*** | ***M*** | ***SD*** | ***lower*** | ***upper*** |
| Intercept | 1.00 | 0.00 | 1.00 | 0.00 | 1.00 | 9.24 | 0.37 | 8.59 | 9.98 |
| BIS Total | 0.50 | 0.50 | 0.10 | 0.87 | 0.15 | 0.00 | 0.05 | -0.14 | 0.05 |
| BAS-FS | 0.50 | 0.50 | 0.23 | 0.77 | 0.29 | 0.08 | 0.20 | 0.00 | 0.59 |
| BAS-D | 0.50 | 0.50 | 0.64 | 0.36 | 1.80 | 0.48 | 0.44 | 0.00 | 1.19 |
| BAS-RR | 0.50 | 0.50 | 0.20 | 0.80 | 0.25 | -0.07 | 0.23 | -0.75 | 0.08 |

*Notes.* -FS, Fun Seeking; -D, Drive; -RR, Reward Responsiveness.

**Table S3.2.12.** Posterior summaries of regression coefficients with regard to the iWCST variable nPE% and PHQ-4 scores

|  | | | | | | | | **95% Credible Interval** | |
| --- | --- | --- | --- | --- | --- | --- | --- | --- | --- |
| **Coefficient** | ***p*(incl)** | ***p(*excl)** | ***p*(incl\|data)** | ***p*(excl\|data)** | ***BF_inclusion_*** | ***M*** | ***SD*** | ***lower*** | ***upper*** |
| Intercept | 1.00 | 0.00 | 1.00 | 0.00 | 1.00 | 9.24 | 0.37 | 8.54 | 9.97 |
| PHQ Depression | 0.50 | 0.50 | 0.11 | 0.89 | 0.12 | 0.00 | 0.10 | -0.14 | 0.32 |
| PHQ Anxiety | 0.50 | 0.50 | 0.19 | 0.81 | 0.23 | 0.06 | 0.15 | 0.00 | 0.49 |

**Table S3.2.13.** Bayesian model comparison for the iWCST variable rPE%

| **Models** | ***p*(M)** | ***p*(M\|data)** | ***BF_M_*** | ***BF_10_*** | ***R²*** | ***VIF*** |
| --- | --- | --- | --- | --- | --- | --- |
| BFI-10 |  |  |  |  |  |  |
| Null model | 0.03 | 0.53 | 34.55 | 1.00 | 0.00 |  |
| Neuroticism | 0.03 | 0.07 | 2.48 | 0.14 | 0.00 | 1.03 |
| Extroversion | 0.03 | 0.07 | 2.39 | 0.14 | 0.00 | 1.02 |
| Agreeableness | 0.03 | 0.06 | 2.07 | 0.12 | 0.00 | 1.01 |
| Conscientiousness | 0.03 | 0.06 | 1.89 | 0.11 | 0.00 | 1.03 |
| Openness | 0.03 | 0.05 | 1.78 | 0.10 | 0.00 | 1.02 |
| BIS/BAS-11 |  |  |  |  |  |  |
| Null model | 0.06 | 0.65 | 27.57 | 1.00 | 0.00 |  |
| BIS Total | 0.06 | 0.07 | 1.15 | 0.11 | 0.00 | 1.01 |
| BAS-FS | 0.06 | 0.07 | 1.13 | 0.11 | 0.00 | 1.01 |
| BAS-RR | 0.06 | 0.07 | 1.11 | 0.11 | 0.00 | 1.19 |
| BAS-D | 0.06 | 0.07 | 1.04 | 0.10 | 0.00 | 1.20 |
| PHQ-4 |  |  |  |  |  |  |
| Null model | 0.25 | 0.68 | 6.32 | 1.00 | 0.00 |  |
| PHQ Anxiety | 0.25 | 0.18 | 0.64 | 0.26 | 0.00 | 1.71 |
| PHQ Depression | 0.25 | 0.12 | 0.40 | 0.17 | 0.00 | 1.71 |

*Notes.* The table presents results only for the null model and single predictor models for simplicity. These models were chosen to assess the contribution of individual predictors. VIF, variance inflation factor, calculated in classical linear regression as suggested by van den Bergh et al. (2021).

**Table S3.2.14.** Posterior summaries of regression coefficients with regard to the iWCST variable rPE% and BFI-10 scores

|  | | | | | | | | **95% Credible Interval** | |
| --- | --- | --- | --- | --- | --- | --- | --- | --- | --- |
| **Coefficient** | ***p*(incl)** | ***p(*excl)** | ***p*(incl\|data)** | ***p*(excl\|data)** | ***BF_inclusion_*** | ***M*** | ***SD*** | ***lower*** | ***upper*** |
| Intercept | 1.00 | 0.00 | 1.00 | 0.00 | 1.00 | 3.94 | 0.24 | 3.47 | 4.39 |
| Extroversion | 0.50 | 0.50 | 0.15 | 0.85 | 0.17 | -0.03 | 0.12 | -0.46 | 0.11 |
| Agreeableness | 0.50 | 0.50 | 0.13 | 0.87 | 0.15 | -0.01 | 0.07 | -0.30 | 0.04 |
| Conscientiousness | 0.50 | 0.50 | 0.12 | 0.88 | 0.14 | -0.01 | 0.07 | -0.26 | 0.08 |
| Neuroticism | 0.50 | 0.50 | 0.15 | 0.85 | 0.18 | 0.02 | 0.09 | -0.01 | 0.33 |
| Openness | 0.50 | 0.50 | 0.12 | 0.88 | 0.13 | 0.00 | 0.06 | -0.21 | 0.03 |

**Table S3.2.15.** Posterior summaries of regression coefficients with regard to the iWCST variable rPE% and BIS/BAS-11 scores

|  | | | | | | | | **95% Credible Interval** | |
| --- | --- | --- | --- | --- | --- | --- | --- | --- | --- |
| **Coefficient** | ***p*(incl)** | ***p(*excl)** | ***p*(incl\|data)** | ***p*(excl\|data)** | ***BF_inclusion_*** | ***M*** | ***SD*** | ***lower*** | ***upper*** |
| Intercept | 1.00 | 0.00 | 1.00 | 0.00 | 1.00 | 3.94 | 0.24 | 3.47 | 4.40 |
| BIS Total | 0.50 | 0.50 | 0.11 | 0.89 | 0.13 | 0.00 | 0.03 | -0.09 | 0.03 |
| BAS-FS | 0.50 | 0.50 | 0.11 | 0.89 | 0.13 | 0.01 | 0.07 | -0.00 | 0.21 |
| BAS-D | 0.50 | 0.50 | 0.11 | 0.90 | 0.12 | 0.01 | 0.07 | -0.05 | 0.20 |
| BAS-RR | 0.50 | 0.50 | 0.11 | 0.89 | 0.12 | -0.01 | 0.08 | -0.18 | 0.10 |

*Notes.* -FS, Fun Seeking; -D, Drive; -RR, Reward Responsiveness.

**Table S3.2.16.** Posterior summaries of regression coefficients with regard to the iWCST variable rPE% and PHQ-4 scores

|  | | | | | | | | **95% Credible Interval** | |
| --- | --- | --- | --- | --- | --- | --- | --- | --- | --- |
| **Coefficient** | ***p*(incl)** | ***p(*excl)** | ***p*(incl\|data)** | ***p*(excl\|data)** | ***BF_inclusion_*** | ***M*** | ***SD*** | ***lower*** | ***upper*** |
| Intercept | 1.00 | 0.00 | 1.00 | 0.00 | 1.00 | 3.94 | 0.24 | 3.42 | 4.37 |
| PHQ Depression | 0.50 | 0.50 | 0.15 | 0.85 | 0.17 | -0.02 | 0.09 | -0.34 | 0.00 |
| PHQ Anxiety | 0.50 | 0.50 | 0.20 | 0.80 | 0.26 | -0.04 | 0.10 | -0.33 | 0.00 |

**Table S3.2.17.** Bayesian model comparison for the iWCST variable IE%

| **Models** | ***p*(M)** | ***p*(M\|data)** | ***BF_M_*** | ***BF_10_*** | ***R²*** | ***VIF*** |
| --- | --- | --- | --- | --- | --- | --- |
| BFI-10 |  |  |  |  |  |  |
| Null model | 0.03 | 0.21 | 8.42 | 1.00 | 0.0 |  |
| Openness | 0.03 | 0.35 | 16.94 | 1.65 | 0.01 | 1.02 |
| Conscientiousness | 0.03 | 0.02 | 0.76 | 0.11 | 0.00 | 1.03 |
| Agreeableness | 0.03 | 0.02 | 0.72 | 0.11 | 0.00 | 1.01 |
| Extroversion | 0.03 | 0.02 | 0.70 | 0.10 | 0.00 | 1.02 |
| Neuroticism | 0.03 | 0.02 | 0.68 | 0.10 | 0.00 | 1.03 |
| BIS/BAS-11 |  |  |  |  |  |  |
| Null model | 0.06 | 0.01 | 0.22 | 1.00 | 0.00 |  |
| BIS Total | 0.06 | 0.26 | 5.21 | 17.80 | 0.02 | 1.01 |
| BAS-D | 0.06 | 0.01 | 0.08 | 0.36 | 0.01 | 1.19 |
| BAS-FS | 0.06 | 0.01 | 0.07 | 0.32 | 0.01 | 1.01 |
| BAS-RR | 0.06 | 0.00 | 0.04 | 0.16 | 0.00 | 1.19 |
| PHQ-4 |  |  |  |  |  |  |
| Null model | 0.25 | 0.78 | 10.50 | 1.00 | 0.00 |  |
| PHQ Depression | 0.25 | 0.12 | 0.40 | 0.15 | 0.00 | 1.71 |
| PHQ Anxiety | 0.25 | 0.09 | 0.28 | 0.11 | 0.00 | 1.71 |

*Notes.* The table presents results only for the null model and single predictor models for simplicity. These models were chosen to assess the contribution of individual predictors. *N* = 487 due to two missing values for the iWCST variable IE%. VIF, variance inflation factor, calculated in classical linear regression as suggested by van den Bergh et al. (2021).**Table S3.2.18.** Posterior summaries of regression coefficients with regard to the iWCST variable IE% and BFI-10 scores

|  | | | | | | | | **95% Credible Interval** | |
| --- | --- | --- | --- | --- | --- | --- | --- | --- | --- |
| **Coefficient** | ***p*(incl)** | ***p(*excl)** | ***p*(incl\|data)** | ***p*(excl\|data)** | ***BF_inclusion_*** | ***M*** | ***SD*** | ***lower*** | ***upper*** |
| Intercept | 1.00 | 0.00 | 1.00 | 0.00 | 1.00 | 14.93 | 0.62 | 13.70 | 16.15 |
| Extroversion | 0.50 | 0.50 | 0.14 | 0.86 | 0.16 | 0.03 | 0.26 | -0.60 | 0.58 |
| Agreeableness | 0.50 | 0.50 | 0.14 | 0.86 | 0.16 | -0.02 | 0.18 | -0.64 | 0.15 |
| Conscientiousness | 0.50 | 0.50 | 0.15 | 0.85 | 0.18 | -0.04 | 0.22 | -0.91 | 0.15 |
| Neuroticism | 0.50 | 0.50 | 0.13 | 0.87 | 0.15 | 0.01 | 0.18 | -0.21 | 0.61 |
| Openness | 0.50 | 0.50 | 0.67 | 0.33 | 2.04 | 0.75 | 0.65 | 0.00 | 1.80 |

**Table S3.2.19.** Posterior summaries of regression coefficients with regard to the iWCST variable IE% and BIS/BAS-11 scores

|  | | | | | | | | **95% Credible Interval** | |
| --- | --- | --- | --- | --- | --- | --- | --- | --- | --- |
| **Coefficient** | ***p*(incl)** | ***p(*excl)** | ***p*(incl\|data)** | ***p*(excl\|data)** | ***BF_inclusion_*** | ***M*** | ***SD*** | ***lower*** | ***upper*** |
| Intercept | 1.00 | 0.00 | 1.00 | 0.00 | 1.00 | 14.93 | 0.62 | 13.78 | 16.15 |
| BIS Total | 0.50 | 0.50 | 0.97 | 0.03 | 27.74 | -0.68 | 0.25 | -1.22 | -0.23 |
| BAS-FS | 0.50 | 0.50 | 0.35 | 0.65 | 0.54 | -0.26 | 0.46 | -1.48 | 0.07 |
| BAS-D | 0.50 | 0.50 | 0.50 | 0.50 | 0.99 | 0.51 | 0.65 | -0.09 | 1.90 |
| BAS-RR | 0.50 | 0.50 | 0.28 | 0.72 | 0.38 | -0.23 | 0.51 | -1.72 | 0.09 |

*Notes.* -FS, Fun Seeking; -D, Drive; -RR, Reward Responsiveness.

**Table S3.2.20.** Posterior summaries of regression coefficients with regard to the iWCST variable IE% and PHQ-4 scores

|  | | | | | | | | **95% Credible Interval** | |
| --- | --- | --- | --- | --- | --- | --- | --- | --- | --- |
| **Coefficient** | ***p*(incl)** | ***p(*excl)** | ***p*(incl\|data)** | ***p*(excl\|data)** | ***BF_inclusion_*** | ***M*** | ***SD*** | ***lower*** | ***upper*** |
| Intercept | 1.00 | 0.00 | 1.00 | 0.00 | 1.00 | 14.93 | 0.63 | 13.80 | 16.21 |
| PHQ Depression | 0.50 | 0.50 | 0.14 | 0.86 | 0.16 | 0.06 | 0.22 | -0.09 | 0.70 |
| PHQ Anxiety | 0.50 | 0.50 | 0.10 | 0.90 | 0.12 | 0.01 | 0.14 | -0.10 | 0.47 |
